# Stimulation selectively blocks subthalamic high β–cortical high γ coupling in Parkinson’s disease

**DOI:** 10.1016/j.neurot.2026.e01059

**Published:** 2026-09-04

**Authors:** Ádám József Berki, Hao Ding, Marcell Palotai, László Halász, Loránd Erőss, Gábor Fekete, László Bognár, Andrea Kelemen, Borbála Jávor-Duray, Éva Pichner, Avin Aphrodite Babakhani, Muthuraman Muthuraman, Gertrúd Tamás

**Affiliations:** a Department of Neurology, Semmelweis University, Balassa utca 6, Budapest, 1083, Hungary; b Department of Neurology, Julius-Maximilians-Universität of Würzburg, Josef-Schneider Sraße 11, Würzburg, 97080, Germany; c Department of Neurosurgery and Neurointervention, Semmelweis University, Laky Adolf út 44, Budapest, 1145, Hungary; d Department of Neurosurgery, University of Debrecen, Móricz Zsigmond út 22, Debrecen, 4032, Hungary; e Department of Neurology, Bajcsy-Zsilinszky Hospital and Clinic, Maglódi út 89-91, Budapest, 1106, Hungary; f János Szentágothai Doctoral School of Neurosciences, Semmelweis University, Üllői út 26, Budapest, Hungary; g Athinoula A. Martinos Center for Biomedical Imaging, Boston, 149 13th St, Charlestown, MA, 02129, USA; h Department of Neurology, Massachusetts General Hospital, 15 Parkman St #835, Boston, MA, 02114, USA; i Informatics for Medical Technology, University of Augsburg, Eichleitnerstraße 30, Augsburg, 86159, Germany

**Keywords:** Parkinson’s disease, Subthalamic stimulation, Hyperdirect pathway, Beta band, Gamma band, Phase-amplitude coupling

## Abstract

Subthalamic stimulation in Parkinson’s disease may operate by suppressing high-beta connectivity along the hyperdirect pathway and by promoting gamma motor cortical processing. We examined how the phase of beta activity shapes the amplitude of the gamma rhythm along hyperdirect connections and how stimulation influences this coupling. Thirty-eight patients with akinetic-rigid Parkinson’s disease treated with bilateral subthalamic stimulation were recruited. A high-density electroencephalogram was recorded at rest and while patients drew self-paced and traced spirals on a digital tablet at four stimulation levels. We analyzed time-resolved phase-amplitude coupling between low (13–20 Hz) and high beta (21–30 Hz) and low (31–60 Hz) and high gamma (61–100 Hz) frequency band pairs between the subthalamic nucleus and motor cortical areas. The stimulation-induced decreases in phase-amplitude coupling were correlated with real-time improvement in bradykinesia and predicted by clinical factors. Among the subthalamic beta–cortical gamma bands, the high beta–high gamma phase-amplitude coupling was the largest (p < 0.001), and decreased the most at the highest stimulation level (p < 0.001), in correlation with the improvement in bradykinesia. Its stimulation-induced decrease could be predicted in task-specific pathways consisting of the primary motor cortex and the dorsal premotor cortex; the rate of improvement in drawing speed and the active contact locations were the key predictors. Phase–amplitude coupling of the cortical beta–subthalamic gamma band pairs did not respond to stimulation. Subthalamic stimulation selectively interferes with the subthalamic high beta-driven cortical gamma block in task-specific pathways in Parkinson’s disease, tracking the improvements in bradykinesia.

## Graphical abstract

**Figure:**
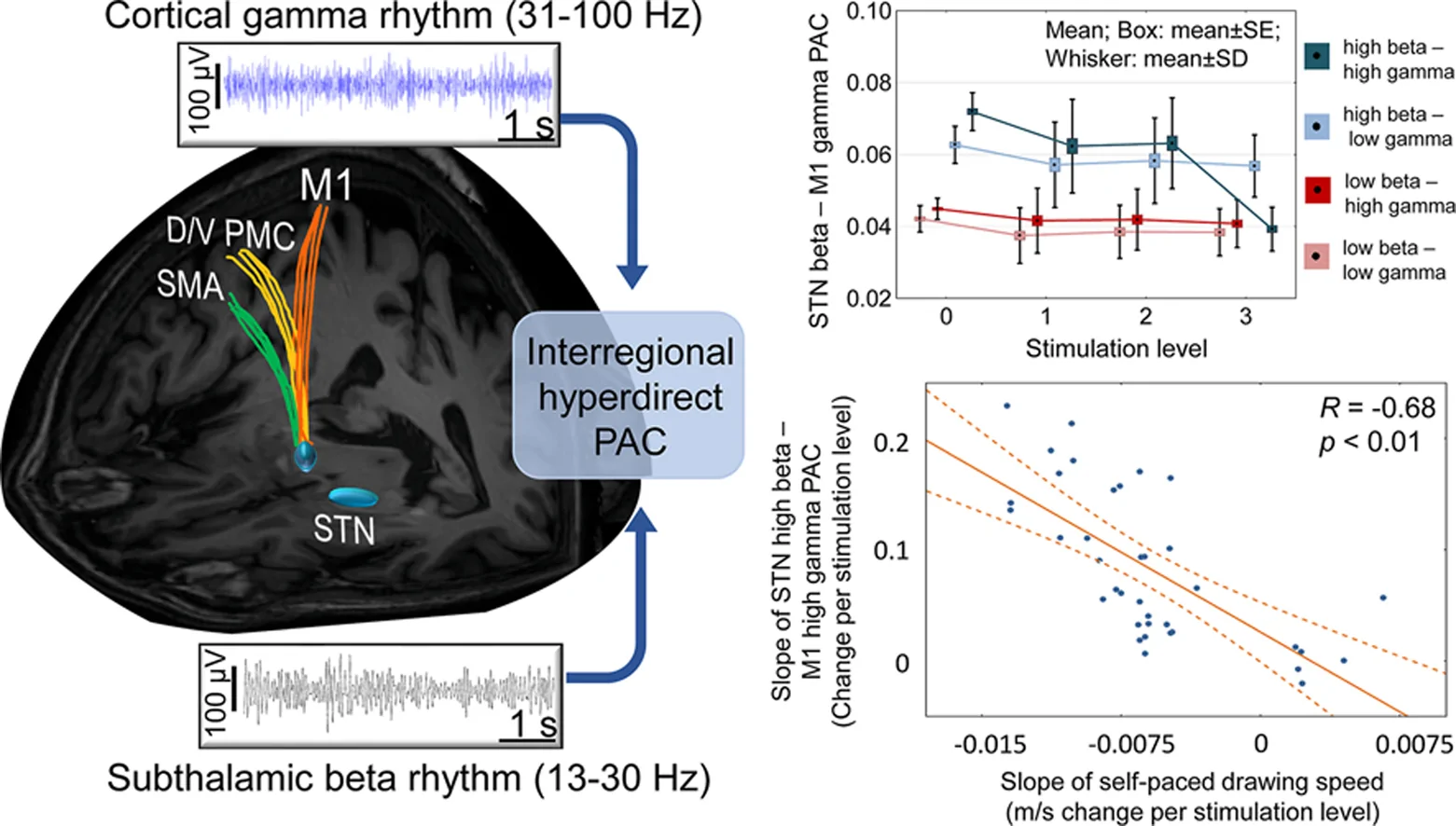

## Introduction

In Parkinson’s disease (PD), excessive phase–amplitude coupling (PAC) between beta oscillations (13–30 Hz) and higher frequencies (>30 Hz) hinders motor circuit dynamics across the basal ganglia–thalamocortical network [1,2]. Accordingly, the phase of pathological beta activity [3] affects the prokinetic gamma amplitude [4,5], resulting in motor dysfunction in the disease. An earlier study demonstrated that high beta (21–30 Hz) synchronized activity between the subthalamic nucleus (STN) and motor cortical areas is reduced due to subthalamic deep brain stimulation (DBS) in a dose-dependent manner, releasing intercortical gamma band communication with improving bradykinesia [6]. Based on these results, it emerges that subthalamic beta–cortical gamma PAC plays a role in the development of bradykinesia, and it remains unclear how subthalamic stimulation influences it.

Excessive beta–gamma PAC within various anatomical hubs of the motor network and between them has already been explored in Parkinson’s disease.

Intraregional, subcortical PAC was detected within the globus pallidus internus (GPi) [7,8], and the STN [1,[9], [10], [11], [12], [13], [14], [15]]. In the STN (13–20 Hz/17–22 Hz and 230–280 Hz), it was greater contralateral to the more affected hemibody, supporting its link to the pathophysiology of bradykinesia [16]. Intraregional cortical PAC within the primary motor cortex (M1) [15,[17], [18], [19], [20], [21], [22], [23], [24], [25]], premotor cortex [[18], [19], [20],^22^], and the supplementary motor cortex (SMA) [20] were also shown in PD, as well as excessive beta–gamma PAC across different cortical areas in PD [20,26]. Although pathological subthalamo–cortical hyperdirect PAC has also been described in PD [15], its role in motor processing deficits and its reactivity to subthalamic stimulation remain elusive. Moreover, the subthalamo–cortical interregional PAC calculations were often restricted to the STN and M1 [15,27] and did not involve other motor cortex areas. Beta–gamma PAC responded to therapeutic approaches. Levodopa administration decreased it in the STN [1,9,11,14] and within the motor cortical areas [20,21,25]. Subthalamic stimulation reduced PAC in the low beta–(100–400 Hz) [10] band within the STN; in the broad beta– (50–150 [28]/200 [24] Hz) range within the motor cortical areas; however, it was not examined along the STN–motor cortex pathways.

In most studies on subcortical and cortical intraregional beta-gamma PAC, only the resting state [[10], [11], [12],^19^,^20^,^22^,^24^,^28^] was examined; patients performed upper limb movements in some investigations [1,14,18], but only a few of these used EMG or motion sensors [1,14,18] for measurement, rather than UPDRS III scores [12,20,22,28]. The effect of stimulation on PAC values has been studied more frequently in the perioperative phase [11,12,14,22,24], which is affected by the stun effect, than in the chronic phase [28]. Published studies lack individual comparisons of different stimulation levels and did not utilize real-time kinematic analysis of complex hand movements during electrophysiological measurements.

In the present study, we examined patients with Parkinson’s disease in the chronic phase after surgery using a 64-channel EEG in the resting state and while they performed traced and self-paced complex hand movements with their more affected hand at different levels of contralateral stimulation. After reconstructing the original source signals from motor cortical areas and the subthalamic nucleus, we aimed to examine how subthalamic beta activity shapes the amplitude of cortically generated gamma rhythm and how this coupling responds to stimulation ramping. We also tested whether cortical beta–subthalamic gamma PAC may impact bradykinesia and whether it is stimulation-reactive.

## Materials and methods

### Study participants and protocol

**Fig. 1:**
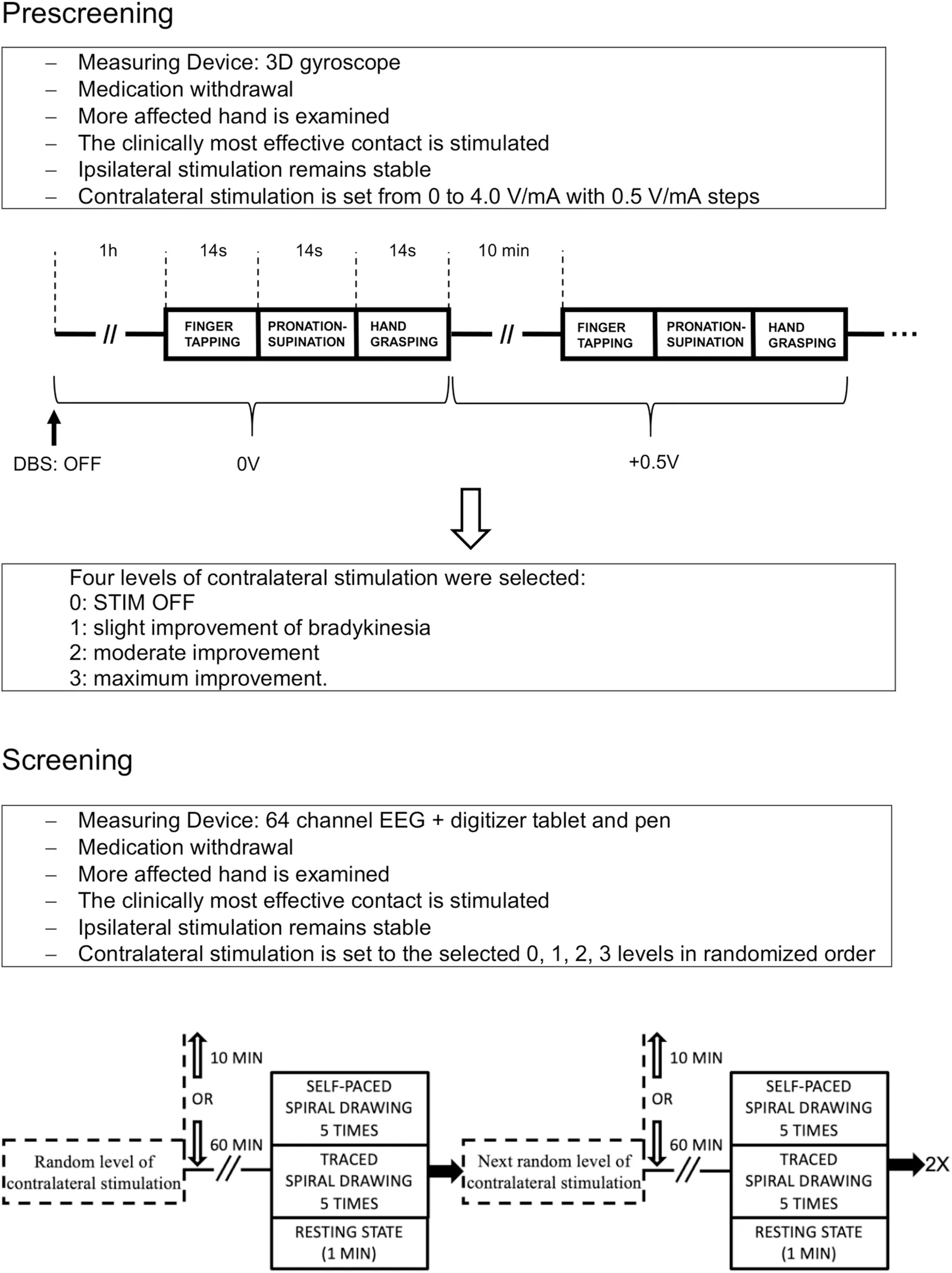
Study protocol.

We enrolled 38 patients with idiopathic akinetic-rigid Parkinson’s disease who had been treated with bilateral STN-DBS for at least 1 year at the Department of Neurology, Semmelweis University, Budapest, Hungary. The Medical Research Council of Hungary approved this study (080958/2015/OTIG), and all patients provided written informed consent. The study was conducted in accordance with the principles of the Declaration of Helsinki. None of the patients had cognitive decline or mood disturbances according to neuropsychological evaluation (Mini-Mental State Examination, Addenbrooke’s Cognitive Examination, Beck’s Depression Inventory). We excluded patients with tremor syndrome and musculoskeletal disorders because these symptoms could bias the PAC estimation [29]. Motor performance was assessed using the Hoehn and Yahr Scale, Unified Parkinson’s Disease Rating Scale Part III (UPDRS III), and Movement Disorders Society–Unified Parkinson’s Disease Rating Scale Part III scores (MDS–UPDRS III). We converted the UPDRS III preoperative scores for 36 of 38 patients and the postoperative scores for 28 of 38 patients to MDS–UPDRS III scores, and used these scores for subsequent analyses. Dopaminergic medications were withdrawn at least 12 h before the test, except for dopamine agonists, for convenience. The study protocol is summarized in Fig. 1.

### Surgical procedure

All patients underwent implantation of bilateral DBS electrodes in the STN using standard stereotactic procedures with the aid of intraoperative microelectrode recordings and clinical testing [30]. Medtronic 3389 leads were implanted in 23 patients, St Jude 6147 in 9 patients, Abbott 6170 in seven patients, and Boston 2202 in one patient.

A detailed description of the surgical procedure has been published previously [31]. The patients had no surgical or hardware-related complications during the perioperative or postoperative phase. After measuring impedance values, stimulation current intensities (mA) were calculated for constant-voltage pulse generators (23 patients).

DBS electrode positions were reconstructed using Lead-DBS software [32] (version 3.1, https://www.lead-dbs.org/). Detailed information can be found in the Supplementary Material.

### Prescreening

We prescreened all patients one day before the primary assessment using the Kinesia motion capture system (Great Lakes Neurotechnologies Inc., USA) to determine four individual stimulation levels with definite symptom improvement (level 0: STIM OFF; level 1: mild improvement; level 2: moderate improvement; level 3: maximum improvement without side effects). Hence, we evaluated the kinematic parameters of finger tapping, hand grasping, and pronation-supination in the most affected hand. First, the contralateral DBS lead was turned off for 1 h. Patients performed each task for 15 s, and then repeated the tasks after stepwise increases in stimulation intensity (0.5 mA increments) until optimal motor performance was achieved without side effects. Kinesia subscores for speed, amplitude, and rhythm were calculated for each patient at each stimulation level [33,34]. These were combined across the three movement tasks to extract the total Kinesia scores (ranging from 0 to 4). Based on these scores, four distinct stimulation levels were selected: 0 (OFF state), 1 (mild improvement), 2 (moderate improvement), and 3 (maximum improvement without side effects). We used the clinically most effective contact location for stimulation with a unipolar configuration for all patients.

### Spiral drawing and analysis

One day after prescreening, we conducted the main measurements following a 12-h medication withdrawal. After resting state measurements, participants sat comfortably at a table and drew Archimedean spirals with their more affected hand (24 patients with right hand and 14 with left hand) on the surface of a digital graphics tablet [Wacom Bamboo Fun Pen and Touch Small Tablet CTH-461, Wacom Technology Corporation, Vancouver, WA; resolution: 2540 lpi (1000 points/cm); sampling rate: 100 Hz; active area: 14.72 × 9.20 cm; and pressure sensitivity: 1024 levels], using a wireless inking pen. Each patient drew five spirals freely (without visual guidance) and five spirals using a template Archimedian spiral (spacing = 1.5 cm; maximal radius covered by the active zone of the tablet = 5.0 cm; three loops) attached to the surface of the tablet. The patients completed the tasks under each of the four preselected stimulation conditions in a counterbalanced order. Patients were instructed to sit with their shoulders parallel to the tablet’s edge and draw spirals at their own pace, starting from the center and moving outward without lifting the pen. When using a template, the active area of the tablet was marked on the paper, and patients were asked not to cross the printed lines.

#### Integrity of the blind

The patients and investigators were blinded to the stimulation levels during measurements. We did not examine their thoughts about the stimulation level that we set during the measurement.

**Fig. 2:**
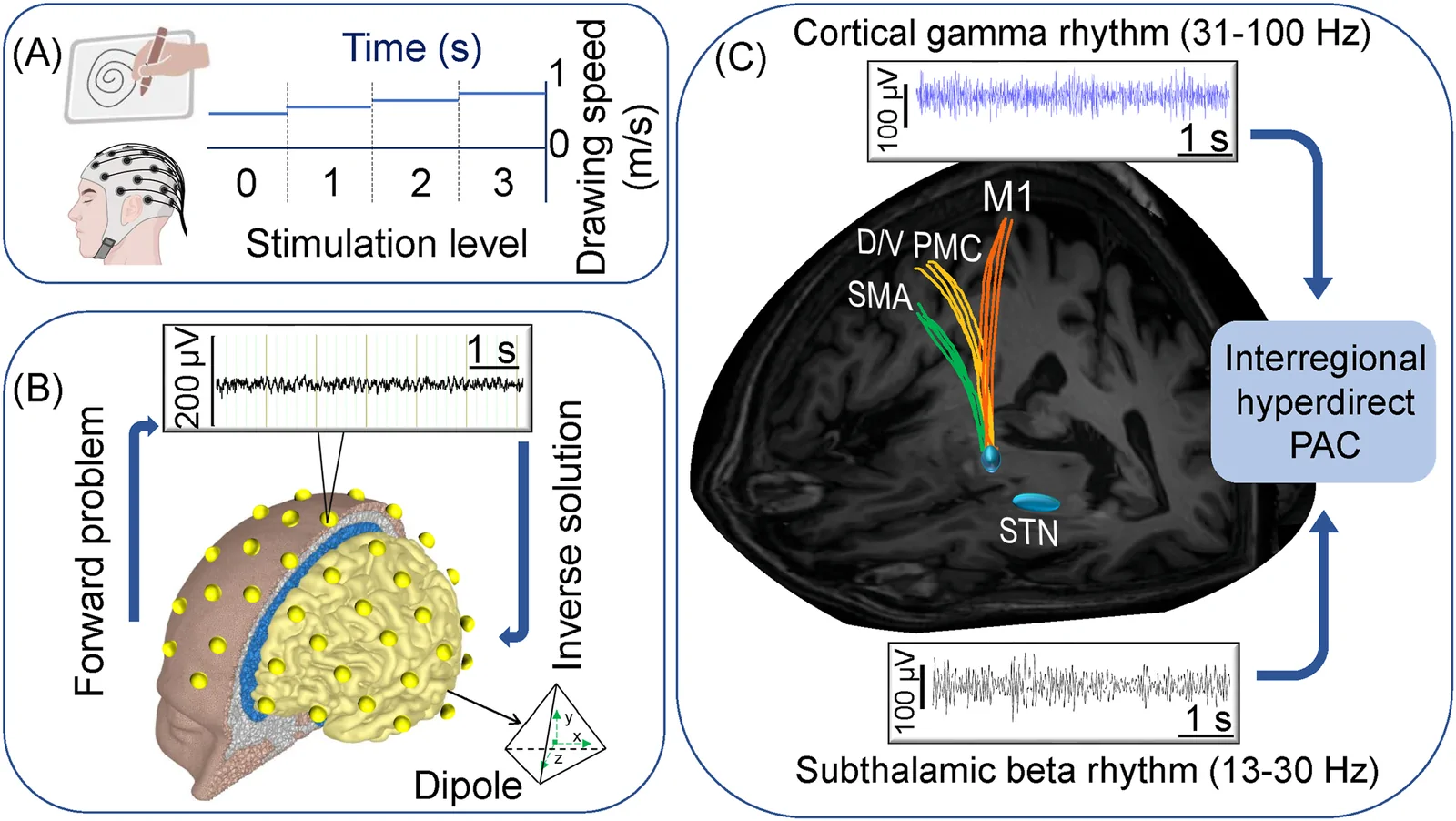
Methodology pipeline. **(A)** A 64-channel EEG was recorded during rest, as well as while patients drew self-paced and traced spirals at four selected stimulation levels with increasing drawing speed. (**B)** Representative cleaned time series after independent component analysis and DBS artifact rejection of the C4 channel, used for coherent neural activity estimation based on a beamformer inverse solution dynamic imaging method. **(C)** Using the estimated source time series, we analyzed time-resolved interregional phase-amplitude coupling along the hyperdirect pathway between subthalamic beta and cortical (primary, supplementary motor, dorsal, and ventral premotor cortex) gamma frequency band pairs. C4: central channel 4, D/V PMC: dorsal and ventral premotor cortices, SMA: supplementary motor area, M1: primary motor cortex, STN: subthalamic nucleus.

Kinematic data points were collected using Neuroglyphics software [35] (http://www.neuroglyphics.org), a Windows-based application designed for recording and analyzing kinematic information. The start and end of each spiral were visually inspected and manually marked during offline analysis. To assess drawing speed, we used mean tangential velocity values derived from the instantaneous X and Y coordinates of the spiral drawing (Fig. 2A). Tangential velocity is the instantaneous linear velocity of an object moving along a circular path.

### EEG recording and preprocessing

We recorded EEG signals during spiral drawing (self-paced and traced spirals) and during a 1-min resting-state condition, separately for each of the four stimulation settings, yielding 4 × 3 EEG files (four stimulation conditions × three task conditions) per patient. Data were collected using a 64-channel EEG system (BrainVision Recorder, Brain Products Co., Munich, Germany) at a sampling rate of 2500 Hz (Fig. 2B). Each recording consisted of the following steps: Initial preprocessing was performed by researchers blinded to the stimulation conditions using MATLAB and the BrainVision Analyzer (Brain Products Co., Munich, Germany). First, EEG data were re-referenced to the common grand-average reference. A fourth-order Butterworth filter was then applied: a high-pass filter (0.5 Hz), a low-pass filter (300 Hz), and notch filters (50, 100, and 150 Hz) to remove line noise. The notch filter has a bandwidth of 5 Hz, which is symmetric around the notch frequency that has been set (i.e., 50 Hz ± 2.5 Hz, or 100 Hz ± 2.5 Hz)*.* Subsequently, semi-automatic independent component analysis (ICA) and inverse ICA were used to eliminate components related to DBS, muscle activity, eye blinks, eye movements, and line-noise artifacts. On average, 6 out of 64 components were removed [6.23 ± 1.19 (mean ± standard deviation)], including DBS components (2 ± 2.23), eye artifacts (1 ± 1.19), line noise (2 ± 0.83), and muscle artifacts (1 ± 0.39). The remaining muscle artifacts were visually inspected, removed, and interpolated using the cubic interpolation method. All channels were used for the analysis. Finally, the EEG data recorded during the drawing of the five spirals were concatenated for subsequent statistical analysis. Source analysis was conducted as described in our previous publication [6] and in the Supplementary Material.

### Power spectral density analysis

To perform spectral analysis, Welch’s periodogram was applied to the artifact-free electroencephalographic (EEG) signals using 1-s Hamming-windowed segments with 50% overlap. Power spectral density was computed for four frequency bands: low beta (13–20 Hz), high beta (21–30 Hz), low gamma (31–60 Hz), and high gamma (61–100 Hz). Power values were calculated separately for each of the five brain regions. Spectral power was quantified for EEG recordings obtained during the resting-state condition, self-paced spiral drawing, and traced spiral drawing, separately for the hemisphere contralateral and ipsilateral to the drawing hand.

### Phase–amplitude coupling analysis

We calculated the inter-regional cross-frequency coupling parameters using the time-resolved phase–amplitude coupling measure implemented in the Brainstorm software [36,37]. This method estimates the dynamics of interconnected slow and fast oscillations using sliding time windows, thereby achieving high temporal resolution and low noise sensitivity.

We determined the relationship between beta activity of the subthalamic nucleus (frequency for phase) and gamma activity of the supplementary motor area, primary motor cortex, and dorsal and ventral premotor cortices (frequency for amplitude). We also investigated the relationship between beta activity in the above-mentioned cortical areas (cortical frequency for phase) and gamma activity in the subthalamic nucleus (frequency for amplitude). For the modulator beta frequency, we used the 13–20 Hz (low beta band) and 21–30 Hz (high beta band) ranges, while modulated gamma frequencies were set at 31–60 Hz (low gamma band) and 61–100 Hz (high gamma band) frequency ranges, resulting in the following frequency-pair combinations: low beta–low gamma, low beta–high gamma, high beta–low gamma, and high beta–high gamma bands. For each patient, task condition, and stimulation setting, we estimated time-resolved PAC. Pre-processed time series were split into 1-s-long epochs and analyzed with a 50% overlap. The overall magnitude of the coupling strength was derived for each frequency pair. Coupling maps were aligned using dynamic time warping (DTW) and grand-averaged using DTW Barycenter Averaging, which iteratively computes a centroid time series that minimizes the DTW distance to all subjects [[38], [39], [40]].

### Slope calculations

We calculated the slopes of stimulation-induced changes in PAC and the drawing speed. The slope in our study represents the rate of change of the outcome variable (e.g., speed or PAC; Y) per individualized stimulation level (X variable) increase. The values were calculated using ordinary least squares linear regression in TIBCO Statistica software. A positive slope (change per stimulation level) means that both values change in the same direction, while a negative slope indicates an inverse relationship, i.e, as X increases, Y decreases.

### Prediction analysis

Prediction analysis was conducted using a Support Vector Regression (SVR) [2.1] model [41] implemented in Python (scikit-learn v1.3) with Bayesian hyperparameter optimization over the Gaussin kernel. We aimed to predict stimulation-induced high beta–high gamma PAC suppression between the STN and motor cortical areas during self-paced and traced spiral acquisition. The following predictors were considered: age, disease duration, pre- and postoperative clinical scores, and kinematic features derived from spiral drawing. To mitigate multicollinearity, features with a Variance Inflation Factor (VIF) exceeding 5 were iteratively removed prior to model fitting. Model performance was evaluated using 5-fold cross-validation; the out-of-fold (OOF) R^2^, which was computed by pooling held-out predictions across all folds. Statistical significance was assessed via permutation testing (10,000 permutations), in which the observed OOF R^2^ was compared against a null distribution obtained by randomly permuting the target labels while keeping the trained model fixed; p-values were estimated using a continuity-corrected formula. Feature importance was quantified using SHAP (Kernel SHapley Additive exPlanations), which treats each feature as a player contributing to the model output. Global feature importance was summarized as the mean absolute SHAP value across observations.

### Statistical analysis

TIBCO Statistica (version 14.0.1) and MATLAB software were used for statistical analyses. Normal distribution of the data was assessed using the Kolmogorov-Smirnov test.

Beta- and gamma-band power spectral densities were analyzed using repeated-measures analysis of variance (ANOVA), with the following within-group effects: REGION (M1, VPMC, DPMC, SMA, and STN), HEMISPHERE (contralateral and ipsilateral to the task), BAND (low beta, high beta, low gamma, and high gamma), TASK (resting state, self-paced and traced spiral drawing), STIMULATION LEVEL (0–3). For post hoc comparisons, Tukey’s test was applied.

We performed separate repeated measures ANOVA for the subthalamic beta–cortical gamma and the cortical beta–subthalamic gamma PAC values with the following within-group effects: TASK (resting state, self-paced, and traced spiral drawing), BAND PAIR (low beta–low gamma, low beta–high gamma, high beta–low gamma, and high beta–high gamma), REGION PAIR (STN–M1, STN–SMA, STN–DPMC, and STN–VPMC; and, conversely, for the cortico-subthalamic PAC statistics), and STIMULATION LEVEL (0–3). Tukey’s test was used for post hoc comparisons. Tangential velocity, as a measure of drawing speed, was compared across the four stimulation levels, and repeated-measures ANOVA was performed with TASK and STIMULATION LEVEL as within-subjects factors. The slopes of interregional PAC values and tangential velocity of spiral drawing across stimulation levels were correlated in the two tasks using the Pearson correlation test. To prevent type I errors, we used the Bonferroni correction for multiple correlation analyses and plotted the linear model with 95% confidence intervals after outlier removal. Stimulation effects on the MDS–UPDRS III scores were determined using the Wilcoxon matched-pairs signed rank test. The level of significance was set at p < 0.05.

### Data availability

The dataset for this study can be requested from the corresponding author by qualified researchers, subject to informed consent requirements.

## Results

### Patient demographics and clinical results

Table 1 presents the detailed demographic and clinical characteristics of all participants. We recruited 38 patients (14 females and 24 males) with a mean age of 65.6 years. Their motor symptoms, as assessed by the MDS–UPDRS III, demonstrated significant improvement with subthalamic stimulation under medication (Wilcoxon matched-pairs signed-rank test, p = 0.0011) and during medication withdrawal (p < 0.001). The mean distances with standard deviation of the tested active contact from the center of the dorsolateral STN were X: 1.04 ± 0.66 mm; Y: 2.46 ± 1.33 mm; Z: 1.25 ± 1.1 mm; 3D distance: 0.77 ± 0.76 mm.

**Table 1:** Demographics and disease characteristics.

Mean age (SD)		65.6 (7.38) years
Sex		14 females, 24 males
Disease duration		13.2 (6.67) years
Time after operation		2.7 (2.2) years
Left STN stimulation settings	Amplitude	2.3 (0.59) mA
	Pulse width	59.9 (6.06) μs
	Frequency	134.8 (7.36) Hz
Right STN stimulation settings	Amplitude	2.3 (0.83) mA
	Pulse width	60.7 (7.78) μs
	Frequency	135.2 (7.4) Hz
Tested STN		24 left, 14 right
Preoperative MDS–UPDRS III	MED OFF	42.7 (14.7) points
	MED ON	16.2 (8.56) points
MDS–UPDRS III during the study	STIM ON-MED OFF	16.7 (10.6) points
	STIM ON-MED ON	11.8 (5.70) points
Hoehn-Yahr stage	Preoperative	2.49 (0.82) scores
	At the study	1.32 (0.79) scores
Levodopa equivalent dose	Preoperative	745.3 (540.38) mg
	At the study	407.4 (281.07) mg

SD, standard deviation; STN, subthalamic nucleus; MDS–UPDRS, International Parkinson and Movement Disorder Society–Unified Parkinson’s Disease Rating Scale.

### Beta, gamma power changes due to stimulation

In each region of the two hemispheres, in each task, the power of the beta activity was higher than that of gamma activity (BAND effect: F_3,30_ = 22.08; p < 0.001). There was no difference between the low and high beta power (p = 0.494) and the low and high gamma power (p = 0.054). Stimulation induced a decrease in the beta power band and an increase in the gamma power in both the low and high bands in the treated hemisphere (HEMISPHERE x BAND x STIMULATION LEVEL effect: F_9,90_ = 98.78; p < 0.01; Supplementary Figs. 1–5). The power in the maximum levels 2 and 3 of stimulation did not differ in the low and the high beta band (level 0: p < 0.001; level 1: p < 0.002; level 2: p = 0.98; level 3: p = 0.99).

### Stimulation effect on subthalamo–cortical phase–amplitude coupling and drawing speed

We investigated how phase-amplitude coupling between the subthalamic beta and motor cortical gamma activity is affected by the stimulation intensity across different tasks. A comparison of the four subthalamo–cortical frequency band pairs (low beta–low gamma, low beta–high gamma, high beta–low gamma and high beta–high gamma) showed that PAC values were higher when the modulatory frequency was the high beta band compared to the low beta modulatory band; high beta–high gamma PAC values were the highest (ANOVA for repeated measures, BAND PAIR factor: F_3,36_ = 1408.6, p < 0.001; post hoc comparisons: p < 0.001 between the low beta and high beta determined pairs). Between the pairs of modulatory low beta band, as well as between the pairs of modulatory high beta band, PAC values were higher when the high gamma band was the modulated band and not the low gamma band (p < 0.001 for each comparison). Absolute PAC values did not differ among the four analyzed hyperdirect pathways (REGION PAIR factor: F_3,36_ = 1.004, p = 0.4). It was similar across the rest and movement tasks (TASK factor: F_2,24_ = 3.26, p = 0.055).

**Fig. 3:**
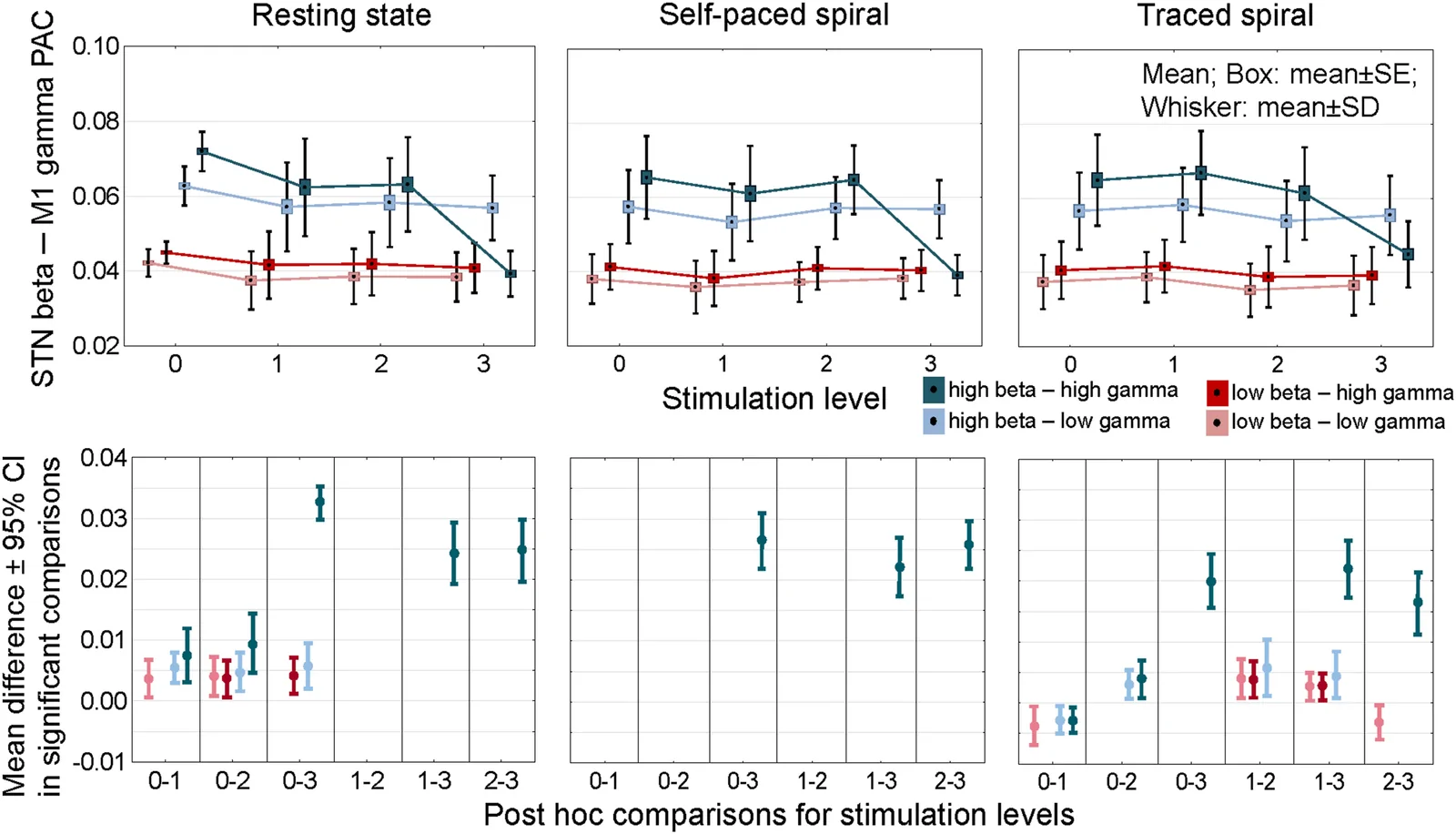
Stimulation effects on STN high beta–M1 high gamma interregional phase–amplitude coupling in PD patients. Subthalamic stimulation decreased the interregional phase-amplitude coupling between the high beta activity of the STN and the high gamma activity of the M1 at the fourth stimulation level in the tested hemisphere during the resting state, self-paced, and voluntary spiral drawing conditions. At the same time, STN-DBS did not influence the coupling between the STN and M1 in the high beta–low gamma, low beta–high gamma, and low beta–low gamma frequency band pairs in either task condition. PAC differences in significant post-hoc comparisons at different stimulation levels are presented in the bottom row. The x-axes of the bottom row plots show the stimulation-level pairs for post hoc comparisons. STN, subthalamic nucleus; M1, primary motor cortex; DBS, deep brain stimulation.

**Fig. 4:**
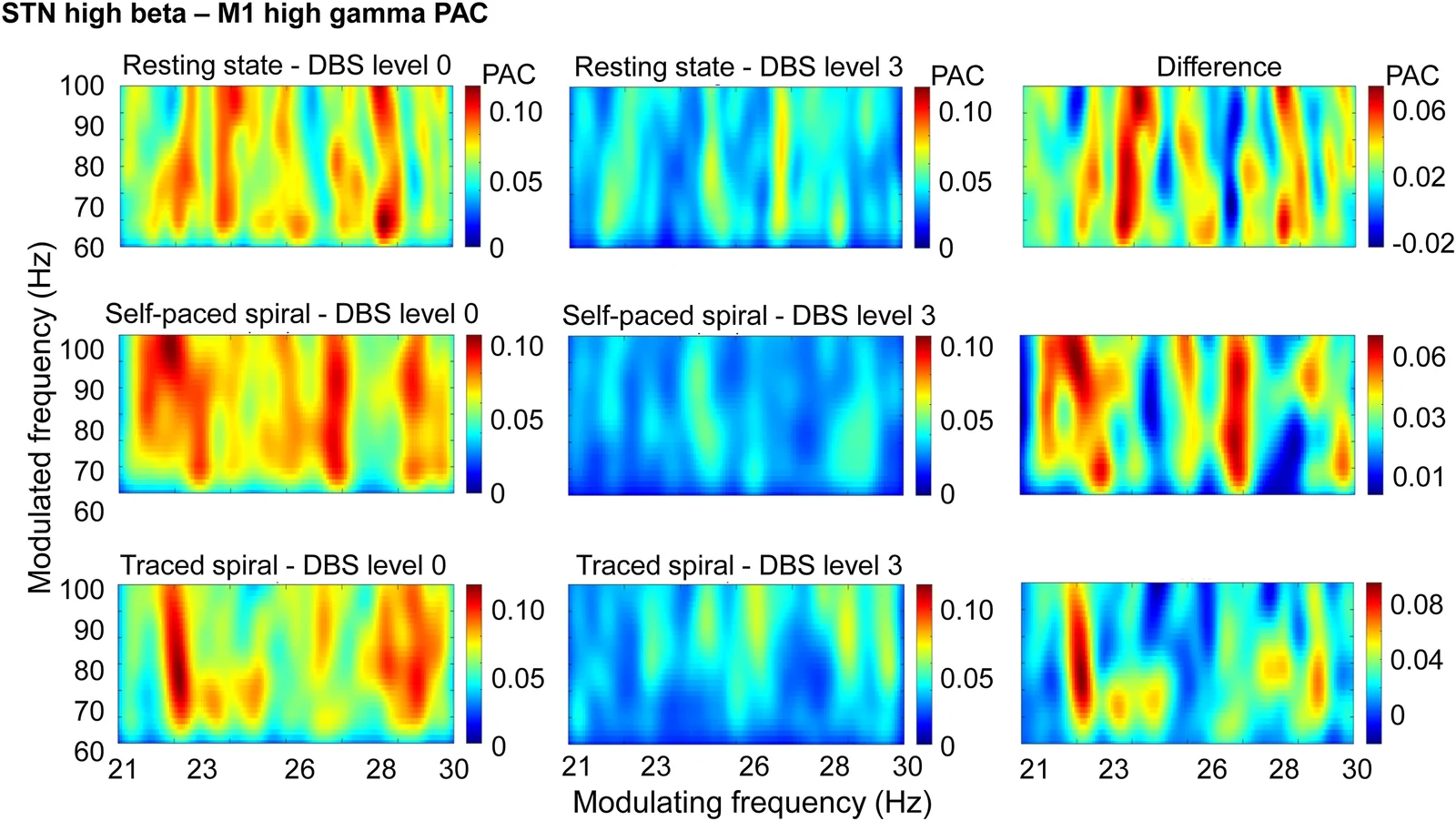
Subthalamic DBS reduces STN high beta and M1 high gamma PAC. Averaged comodulograms of all patients showing the average coupling strength between the phase of the subthalamic high beta band and the amplitude of the M1 high gamma frequency band for each task in the stimulation OFF (level 0) and maximal ON (level 3) stimulation intensity levels. The third column shows the change in PAC values owing to stimulation across a grid of frequencies. PAC was similar in the resting state and during fine motor tasks. At the maximum stimulation level of 3, DBS reduced coupling strength across all task conditions. M1, primary motor cortex; STN, subthalamic nucleus; PAC, time-resolved phase–amplitude coupling; DBS, deep brain stimulation.

Stimulation had a significant effect on absolute PAC values (STIMULATION LEVEL factor: F_3,36_ = 8.038, p < 0.001). When adjusting the stimulation level, the subthalamic high beta–cortical high gamma PAC decreased the most at stimulation level 3 along each analyzed subthalamic-motor cortical pathway (BAND PAIR X STIMULATION LEVEL interaction: F_9,108_ = 248.65, p < 0.001; significant post hoc comparisons determined along each region pair are presented in Fig. 3, Fig. 4 and Supplementary Figs. 6–8).

In the cortical beta–subthalamic gamma PAC analysis, the PAC values were similar across the band pairs and the different hyperdirect pathways during the resting state and the motor tasks. Stimulation had no effect on the absolute cortical beta–subthalamic gamma PAC values (STIMULATION LEVEL factor: F_3,6_ = 0.636, p = 0.6405). Post hoc comparisons for M1-STN PAC determined for each frequency pair and task condition are presented in Supplementary Fig. 9.

We performed further analysis in relation to bradykinesia on the subthalamic beta– cortical gamma PAC values.

As we reported in our previous publication [6], the mean tangential velocity was higher in the self-paced (level 0: 0.57 ± 0.28 m/s; level 1: 0.65 ± 0.30 m/s; level 2: 0.76 ± 0.35 m/s; level 3: 0.81 ± 0.36 m/s) than in the traced spiral drawing (level 0: 0.46 ± 0.21 m/s; level 1: 0.51 ± 0.22 m/s; level 2: 0.55 ± 0.24 m/s; level 3: 0.59 ± 0.26 m/s; ANOVA for repeated measures, TASK factor: F_1,37_ = 33.4, p < 0.001) and significantly increased with increasing stimulation intensity (STIMULATION LEVEL factor: F_3,111_ = 27.43, p < 0.001). The average time needed to draw a single spiral was 14.3 ± 6.42 s, while the average time required to draw 5 spirals was 64.7 ± 23.7 s. After preprocessing, the average concatenated data length was 60.6 ± 19.2 s.

### Correlation between phase–amplitude coupling and task performance slopes

**Fig. 5:**
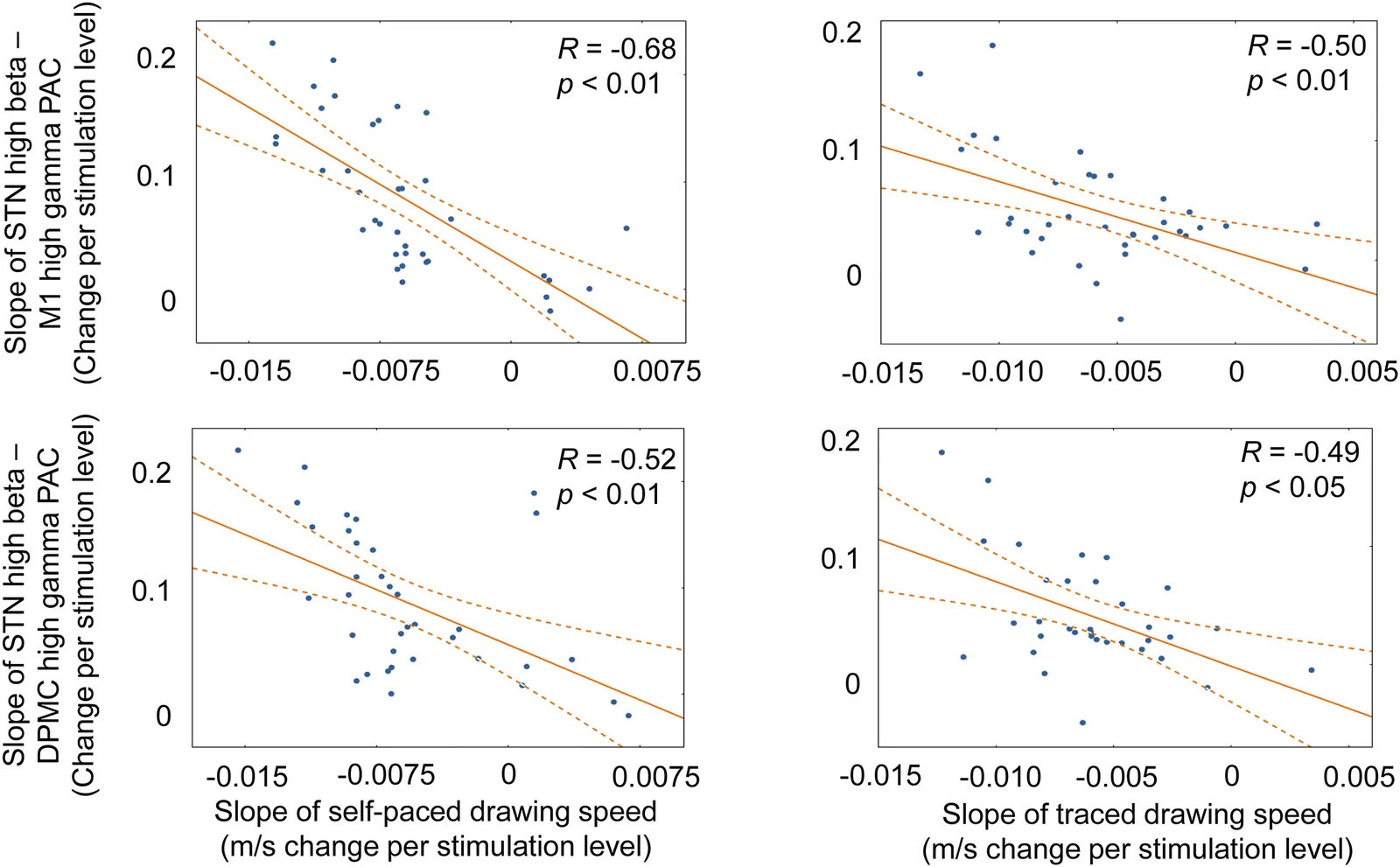
Stimulation-induced improvement of self-paced spiral drawing correlates with the reduction of hyperdirect PAC affecting M1 and DPMC. There was a significant negative correlation between the slope of the stimulation-induced self-paced spiral drawing speed and the subthalamic high beta–cortical high gamma PAC decrease along **(A)** STN–M1 and **(B)** STN–DPMC hyperdirect pathways. Similar to the self-paced spiral drawings, there is a significant negative correlation between the slope of the traced spiral drawing speed and the subthalamic high beta–cortical high gamma PAC decreases along **(C)** STN–M1 and **(D)** STN–DPMC hyperdirect pathways. Solid orange lines represent the best-fit line, whereas dashed lines represent 95% confidence intervals. P values were corrected using the Bonferroni method for multiple comparisons. PAC, interregional phase–amplitude coupling; STN, subthalamic nucleus; M1, primary motor cortex; DPMC, dorsal premotor cortex.

The stimulation-induced increase in self-paced spiral drawing speed showed a significant negative correlation with the decrease in high beta–high gamma phase–amplitude coupling along the STN–M1 (Pearson correlation test, R = −0.68, p < 0.01, Bonferroni corrected, no outliers) and STN–DPMC pathways [R = −0.52, p < 0.01, Bonferroni corrected, without outliers (2 subjects); Fig. 5], while speed improvement did not correlate with PAC decrease along the STN–SMA [R = 0.04, p = 0.80, Bonferroni corrected, without outliers (1 subject)] and STN–VPMC pathways [R = 0.07, p = 0.65, Bonferroni corrected, without outliers (3 subjects); Supplementary Fig. 10].

Similar to the self-paced drawing, stimulation-induced increase in traced spiral drawing speed showed a significant negative correlation only with the decrease in high beta–high gamma phase–amplitude coupling along the STN–M1 [R = −0.50, p < 0.01, Bonferroni corrected, without outliers (1 subject)], and STN–DPMC pathways [R = −0.49, p < 0.05, Bonferroni corrected, without outliers (2 subjects); Fig. 5], and it did not correlate with the decrease in high beta–high gamma coupling strength along the rest of the analyzed pathways: STN–SMA [R = −0.21, p = 0.22, Bonferroni corrected, without outliers (3 subjects)] and STN–VPMC (R = −0.05, p = 0.73, Bonferroni corrected, no outliers, Supplementary Fig. 10).

### Predictors of improving phase–amplitude coupling during stimulation

We aimed to determine whether clinical characteristics might predict stimulation-induced subthalamic high beta–cortical high gamma PAC suppression observed during self-paced and traced spiral acquisition.

**Fig. 6:**
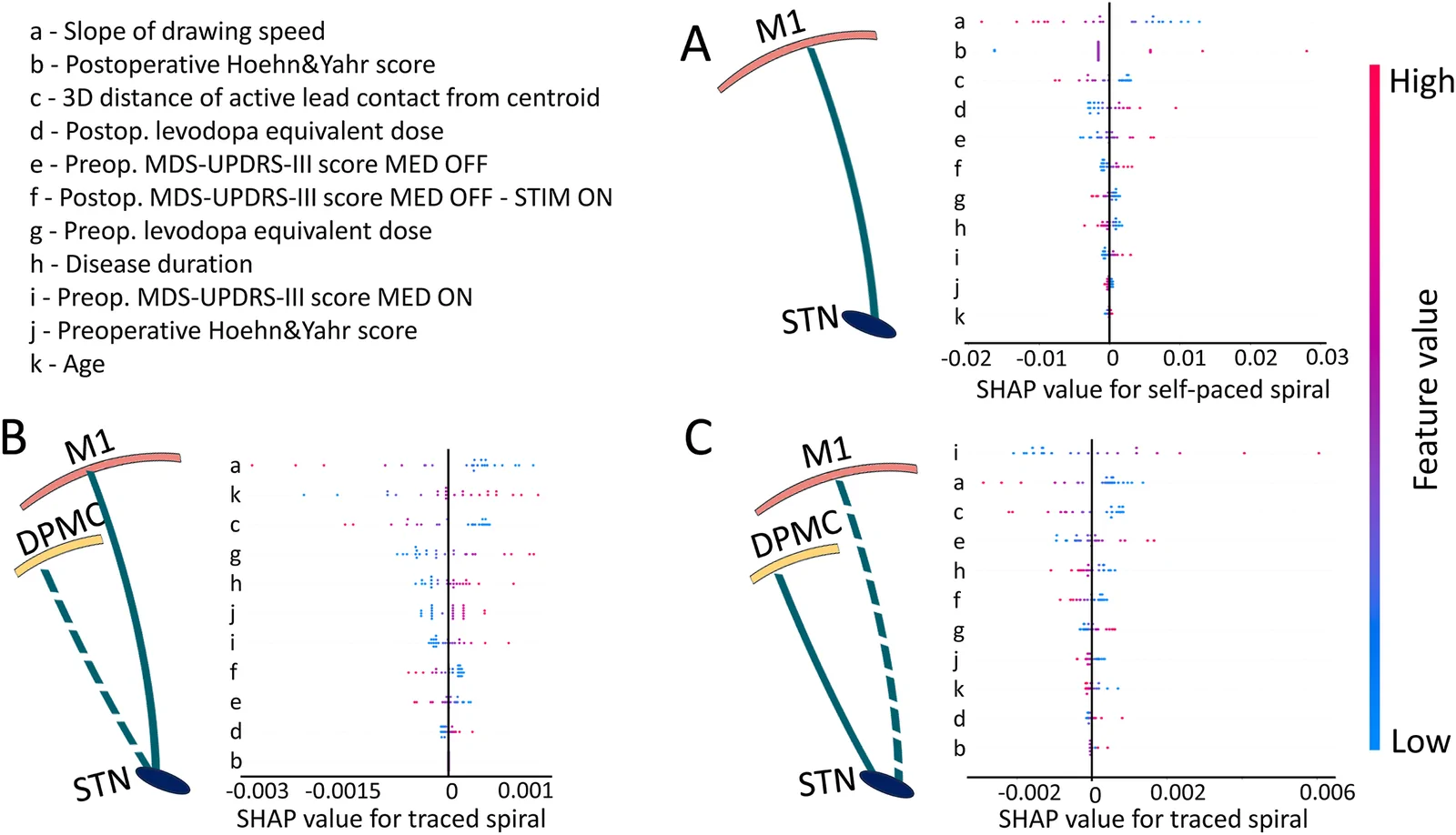
Beeswarm plots for subthalamo–cortical PAC measured during self-paced and traced spiral drawings. The Beeswarm plot shows the significance of each parameter in predicting stimulation-induced changes in the subthalamic high beta–motor cortical high gamma PAC. Along the y-axis, clinical parameters (input variables) were ranked by their mean absolute SHAP values, with the top position indicating greater importance. Clusters of points in red represent high feature values, whereas those in blue illustrate low feature values. The farther a point is from the horizontal axis, the greater its impact on predicting stimulation-induced changes in PAC. **(A)** For STN high beta–M1 high gamma PAC in the self-paced drawing model, the slope of drawing speed, postoperative Hoehn-Yahr scores, and the 3D distance from the subthalamic centroid to the active contact emerged as the most influential features for the support vector regression analysis. **(B)** In the traced drawing model, for STN high beta–M1 high gamma PAC, the slope of drawing speed, the mean age, and the 3D distance from the subthalamic centroid to the active contact influenced the support vector regression the most. **(C)** In the traced drawing model with STN high beta–DPMC high gamma PAC, the preoperative MDS–UPDRS III scores, slope of drawing speed, and 3D distance from the subthalamic centroid to the active contact were the most influential features. STN, subthalamic nucleus; M1, primary motor cortex; DPMC, dorsal premotor cortex; PAC, phase amplitude coupling; SHAP, SHapley Additive exPlanations; MDS–UPDRS, International Parkinson and Movement Disorder Society–Unified Parkinson’s Disease Rating Scale; MED, medication; STIM, stimulation; 3D, three-dimensional.

The Support Vector Regression model to predict high beta–high gamma PAC values along the selected hyperdirect pathways during both self-paced and traced spirals was trained on 11 clinical parameters and the slopes of self-paced and traced drawing speed. In the self-paced spiral drawing task, the model predicted the STN–M1 coupling with a high accuracy level (OOF R^2^ = 0.36; p_perm<0.001), while in the traced drawing task, the STN–DPMC coupling was predicted with the highest (OOF R^2^ = 0.62; p_perm <0.001), and the STN–M1 with lower accuracy (OOF R^2^ = 0.34; p_perm <0.001). Using feature contribution analysis, we identified the most influential features affecting support vector regression predictions of stimulation-induced changes in coupling strength. The slope of the drawing speed emerged as a critical contributor to both the self-paced and traced spirals. In addition, the three-dimensional distance from the subthalamic centroid to the active lead contact was a key factor in both self-paced and traced spiral drawings (Fig. 6).

In contrast, the clinical parameters did not reliably predict the slope of STN–DPMC PAC measured during self-paced spiral drawing or that of STN–VPMC and STN–SMA PAC values measured during both tasks (Supplementary Table 1).

## Discussion

Our results extend our understanding of bradykinesia pathophysiology and the mechanism of action of subthalamic stimulation in Parkinson’s disease. First, we show that the subthalamic beta–cortical gamma PAC is excessive when the high beta is the modulator and the high gamma is the modulated frequency band. Second, hyperdirect subthalamic high beta–cortical high gamma PAC was selectively alleviated by stimulation. Its changes in the M1 and DPMC correlated with improvements in drawing speed during both self-paced and traced movements. Its stimulation-induced changes could also be predicted from the bradykinesia change in task-specific pathways, in a model including the M1 in the self-paced condition and in models incorporating the DPMC and M1 in the traced-movement condition. The active contact location was an influencing factor across all models.

### Subthalamic high beta–cortical high gamma phase–amplitude coupling is prominent

The presence of excessive beta activity and upstream-evolving gamma activity in the subthalamo–cortical network is a hallmark of Parkinson’s disease [42]. High beta activity acts dynamically along the hyperdirect pathways between the STN and M1 [43], PMC [44], and SMA [[43], [44], [45], [46]]. It may be bidirectional [6,43,47]; however, some studies assumed cortical control over subthalamic activity [27,45,46]. High beta oscillations are hampered by active stimulation [6,48], while low beta band activity decreases after levodopa intake [1,49]. The beta–gamma band interaction is complex across the motor network. It was hypothesized that increased basal ganglia synchronization in the beta band might serve as a temporal reference frame for cortical gamma activity through the hyperdirect pathway [3]; therefore, the rhythmic activity in the STN results in fluctuating excitability in cortical neuronal assemblies [50]. In line with the latter, strictly one-directional broadband subthalamic beta–broadband cortical gamma PAC, but not cortical beta–subthalamic gamma PAC, was identified using electrocorticography and implanted STN electrodes in patients with Parkinson’s disease; however, the results suggest bidirectional control over it [15]. In the present study, we identified primary motor and premotor high gamma oscillations, excessively driven by subthalamic high beta activity, in chronically stimulated patients at rest and during complex hand movements. In contrast, cortical beta–subthalamic gamma PAC was neither frequency-specific nor responsive to stimulation.

We could not identify any difference in PAC strength between the self-paced and traced spiral drawings. Similarly, Gong et al. showed that visual feedback did not modulate beta (13–50 Hz)–broadband gamma (50–150 Hz) coupling strength in the sensorimotor cortex, including the M1 and PMC, during slow and fast finger-tapping tasks performed by both patients with PD and healthy controls [18].

The coupling strength was similar at rest and during the movement. Previous results on movement-related behaviors have been inconsistent. Prior research has found a movement-related reduction in both intrasubthalamic and intracortical coupling. Perioperative subthalamic high beta (20–30 Hz)–high frequency oscillation (HFO, 200–400 Hz) PAC displayed a reduction during wrist extension movements [1]. ECoG potentials recorded from the primary motor [15,17,51] and the premotor [51] cortex during a movement task revealed that movement reduces exaggerated PAC between the beta (13–30 Hz) and broadband gamma (50–200 Hz) bands. Additionally, the movement phase was observed to modulate intracortical beta–gamma PAC [29]. An EEG study revealed a broadband beta–(50–150 Hz) gamma PAC rise and decay around the onset and offset of a pressing task in the M1 and PMC [18]. From the results, it was concluded that the lack of stationarity across movement phases may limit the applicability of intracortical beta–gamma PAC as a biomarker for bradykinesia [17,29,51]. As our study is the first to test STN beta–cortical gamma PAC during rest and movement, further studies are needed to confirm that this coupling does not depend on the movement phase and could serve as a potential biomarker of bradykinesia.

### Subthalamic stimulation reduces subthalamic high beta–cortical high gamma hyperdirect PAC

This is the first study to reveal that the subthalamic high beta–cortical high gamma PAC was reactive to stimulation but decreased considerably at stimulation intensities above the second level within its therapeutic window. Individual changes in the M1 and DPMC pathways were correlated with individual motor improvement.

In an earlier whole-brain MEG and STN-LFP study, high beta (20–35 Hz) subthalamic stimulation selectively influenced subthalamic–cortical amplitude coupling, consistent with the clinical outcomes; phase–amplitude coupling was not calculated [52].

### Power analysis

We also calculated power spectral densities in the low beta, high beta, low gamma, and high gamma frequency bands across the four stimulation levels and three task conditions for each analyzed brain region. We found that subthalamic stimulation reduced both low-beta (13–20 Hz) and high-beta (21–30 Hz) power in the STN and motor cortex during spiral drawing. In contrast, low gamma (31–60 Hz) and high gamma (61–100 Hz) power increased in both the STN and motor cortex. Subthalamic stimulation reduced phase-amplitude coupling (PAC) only for the subthalamic high beta–cortical high gamma frequency pairs, suggesting that the reduction in PAC cannot be explained exclusively by alterations in subthalamic beta power. Consistent with our findings, de Hemptinne and colleagues reported that the reduction in intracortical beta–gamma PAC at rest following subthalamic stimulation is not solely attributable to changes in beta or gamma power [17].

### Subthalamic high beta–cortical high gamma PAC operates in task-specific pathways

We demonstrated that the individual increase in self-paced and traced spiral drawing speed negatively correlated with changes in subthalamic high beta–cortical high gamma PAC affecting the M1 and the DPMC.

In the self-paced drawing task, M1 was the participating cortex in a network from which we could predict an STN high beta–M1 high gamma PAC using drawing speed and additional clinical scores. In the traced movement, the DPMC network was the most involved, followed by M1. The primary and premotor cortices are apparent hubs in the beta-driven motor subnetworks [6,19,53]. It has been shown that the dorsal premotor cortex can direct motion based on sensory information [54], as confirmed in a primate model [55] and humans [56]. In contrast, VPMC controls hand movements that involve object manipulation [54,57]. It is associated with more cognitive-related actions, such as understanding motor actions [58].

In our network model, the three-dimensional distance from the subthalamic centroid to the active lead contact was a strong predictor of changes in coupling strength in both self-paced and traced spirals. Hence, this result emphasizes the importance of precise electrode placement during surgery as it influences not only the clinical efficacy but also the reliability of high beta–high gamma hyperdirect PAC as a potential biomarker of bradykinesia in an adaptive subthalamic stimulation system.

### Clinical relevance

These findings provide new insights into the mechanism of action of subthalamic stimulation and pathophysiology of bradykinesia.

To advance adaptive stimulation, our results support a combined subthalamic and motor-cortical multisensing approach. We found that this coupling was not influenced by an ongoing movement program; however, it can be best detected in task-specific hyperdirect pathways. Further studies are needed to confirm the result. A hyperdirect PAC-based neuromodulation strategy would require patient-specific characterization of normal and pathological oscillatory couplings to define therapeutic “eu-PAC” windows. Such characterization may improve offline programming and serve as a feedback signal for next-generation sensing devices and machine-learning-driven algorithms.

### Limitations

The region-of-interest analysis pipeline used in this study included subcortical sources derived from 64-channel EEG data. To optimize the spatial filtering of source signals, we used individual T1 and FEM models. We selected the beta and gamma bands, which showed higher signal-to-noise ratios during a motor task with visual feedback, to obtain optimal source signals.

Although high-density EEG-based source-estimation techniques provide accurate mapping of lateral cortical regions, including the premotor and primary motor cortices, they offer limited sensitivity to mesial cortical and subcortical oscillatory activity. Precise estimation of subthalamic activity would require local field potential analyses, considering the anatomical location and small volume of STN (65–155 mm^3^) [59]. Therefore, in this study we focused exclusively on beta activity (13–30 Hz) from the STN, which has been reliably localized previously using a 64-channel EEG [60,61]. Results of the cortical beta–subthalamic gamma PAC analysis should be interpreted with caution. Nevertheless, validating these results with simultaneous subthalamic and cortical local field potential recordings with a higher signal-to-noise ratio is essential for precisely resolving oscillatory dynamics and further advancing biomarker-guided neuromodulation.

## Author contributions

A.J.B., M.M. and G.T. designed research; M.P., L.H., L.E., G.F., L.B., A.K., B.J-D., E.P. and G.T. performed research; A.J.B., H.D., B.J-D., E.P., A.A.B., M.M. and G.T. analyzed data; and A.J.B., H.D., M.M. and G.T. wrote the paper.

## Declaration of competing interest

The authors declare that they have no known competing financial interests or personal relationships that could have appeared to influence the work reported in this paper.
