# Supplementary material for: Stimulation selectively blocks subthalamic high β–cortical high γ coupling in Parkinson’s disease
Source: Neurotherapeutics. 2026 Sep 4;23(5):e01059. doi: 10.1016/j.neurot.2026.e01059 (PMC13571243; doi:10.1016/j.neurot.2026.e01059)
Supplement: Supplementary file 1 [file mmc1.pdf]

Supplementary material

Berki AJ et al. Stimulation selectively blocks subthalamic high β–cortical high γ coupling in Parkinson's disease

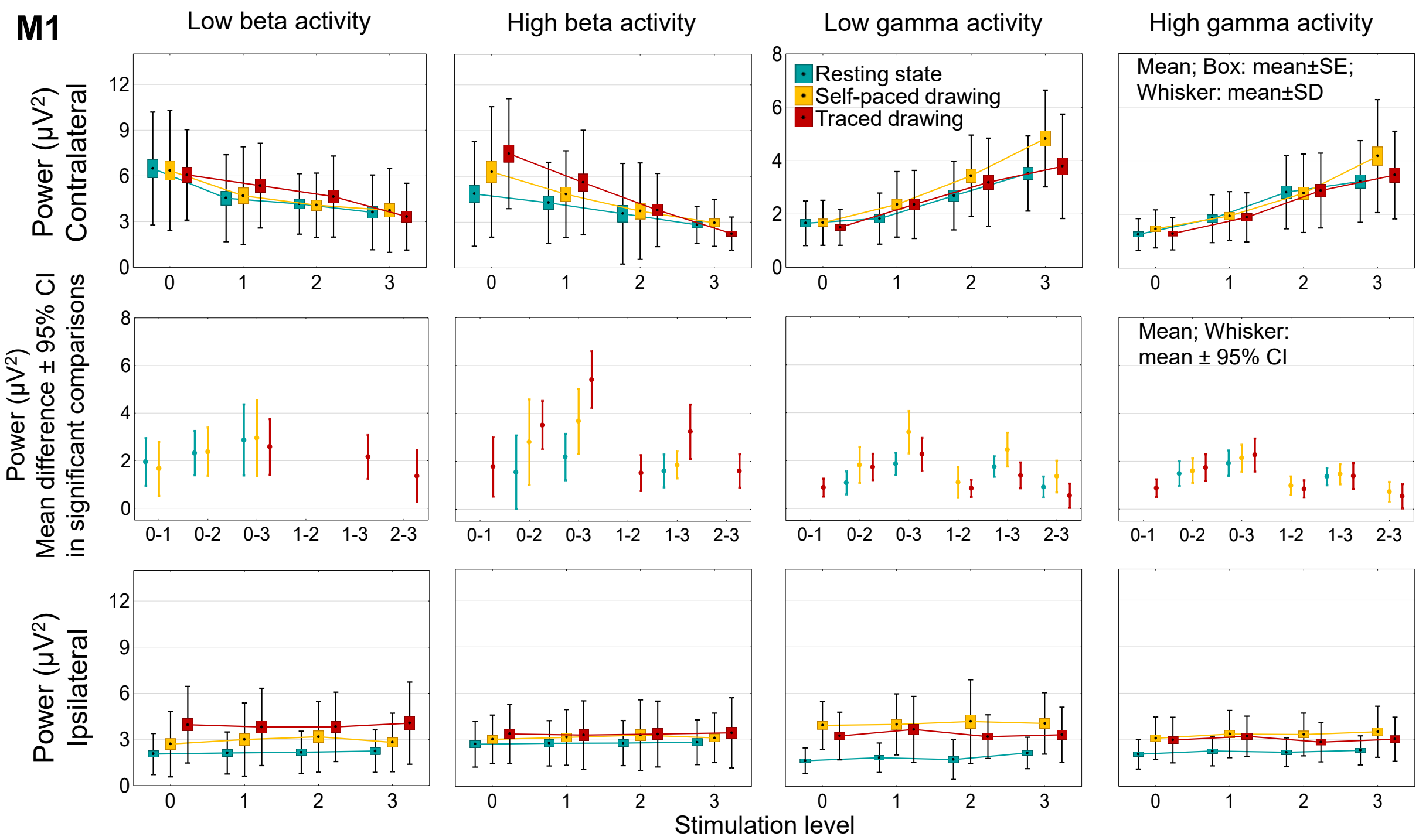

Supplementary Fig. 1. Absolute beta and gamma power changes in the M1

In the tested primary motor cortex, subthalamic stimulation induced a stepwise reduction in absolute low and high beta band power with increasing stimulation intensity during rest and spiral drawing. Conversely, low- and high-gamma band power increased in a stimulation level-dependent manner. Significant post hoc comparisons of stimulation level effects are presented in the middle row. In the ipsilateral (untested) hemisphere, beta and gamma band power remained unchanged across stimulation intensities (bottom row). A subset of the data has been published previously[1]. M1, primary motor cortex

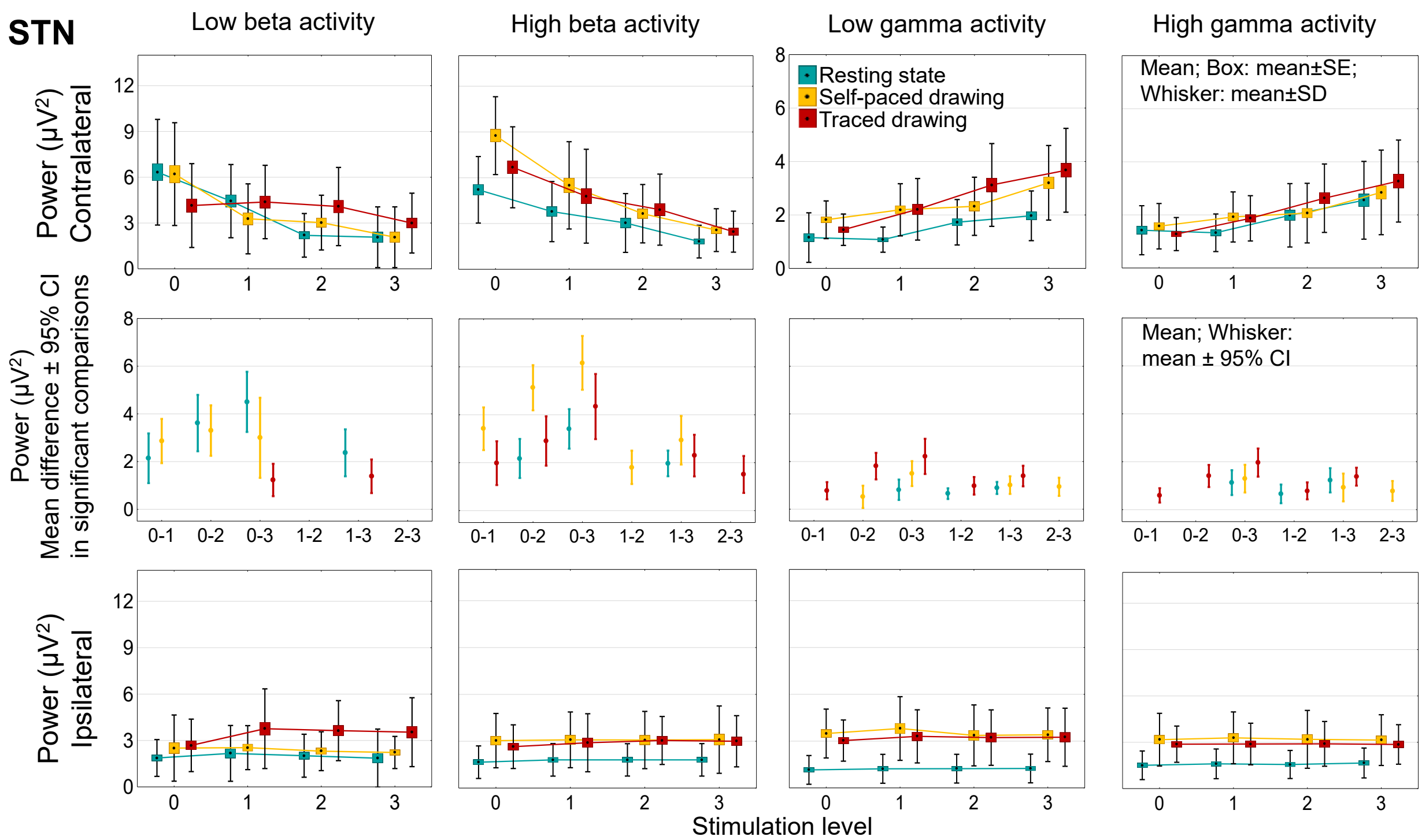

Supplementary Fig. 2. Absolute beta and gamma power changes in the STN

Stimulation induced similar changes in low and high beta and gamma power as in the M1. A subset of the data has been published previously[1]. STN, subthalamic nucleus

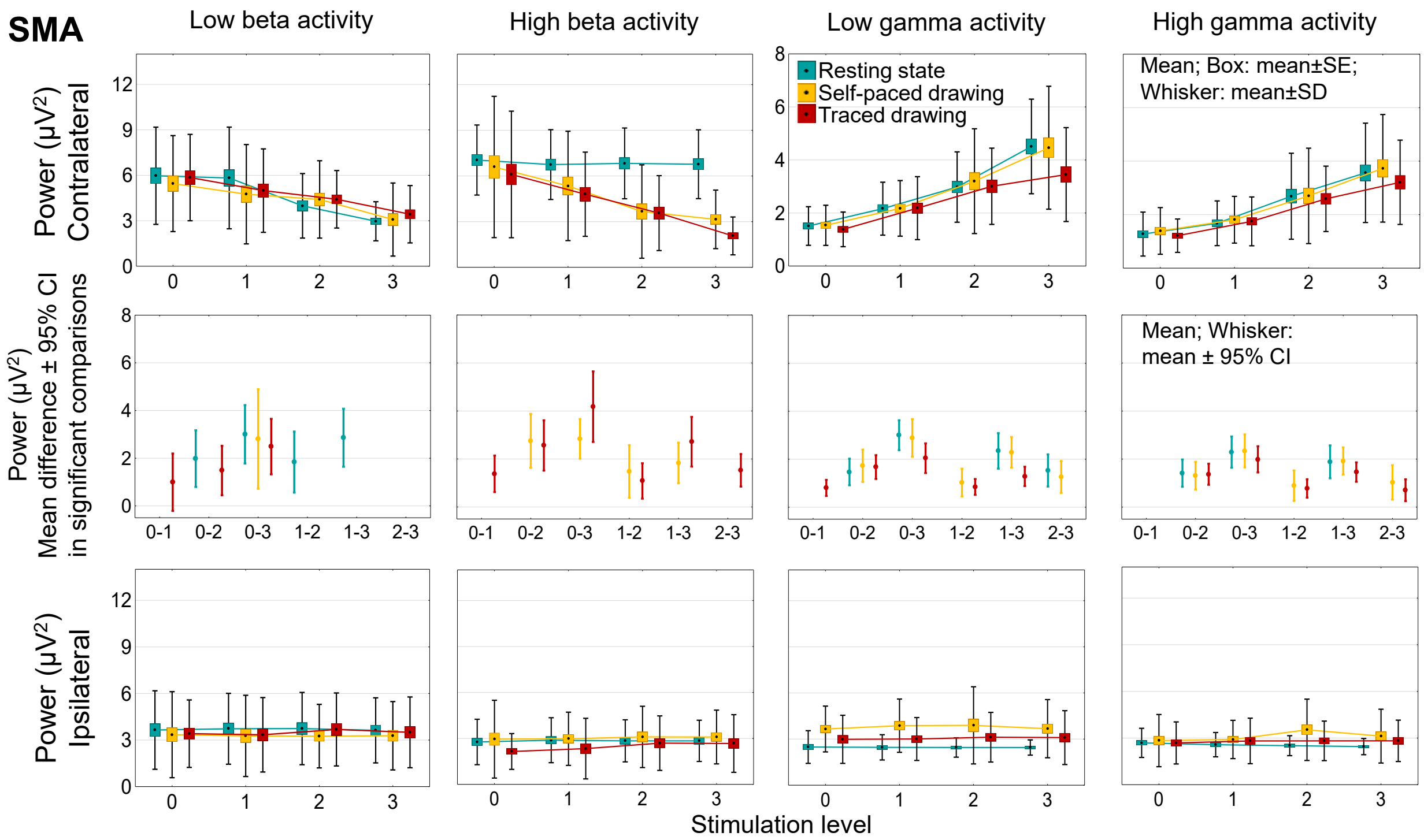

Supplementary Fig. 3. Effects of subthalamic stimulation on absolute beta and gamma band power in the SMA

A subset of the data has been published previously[1]. SMA, supplementary motor cortex

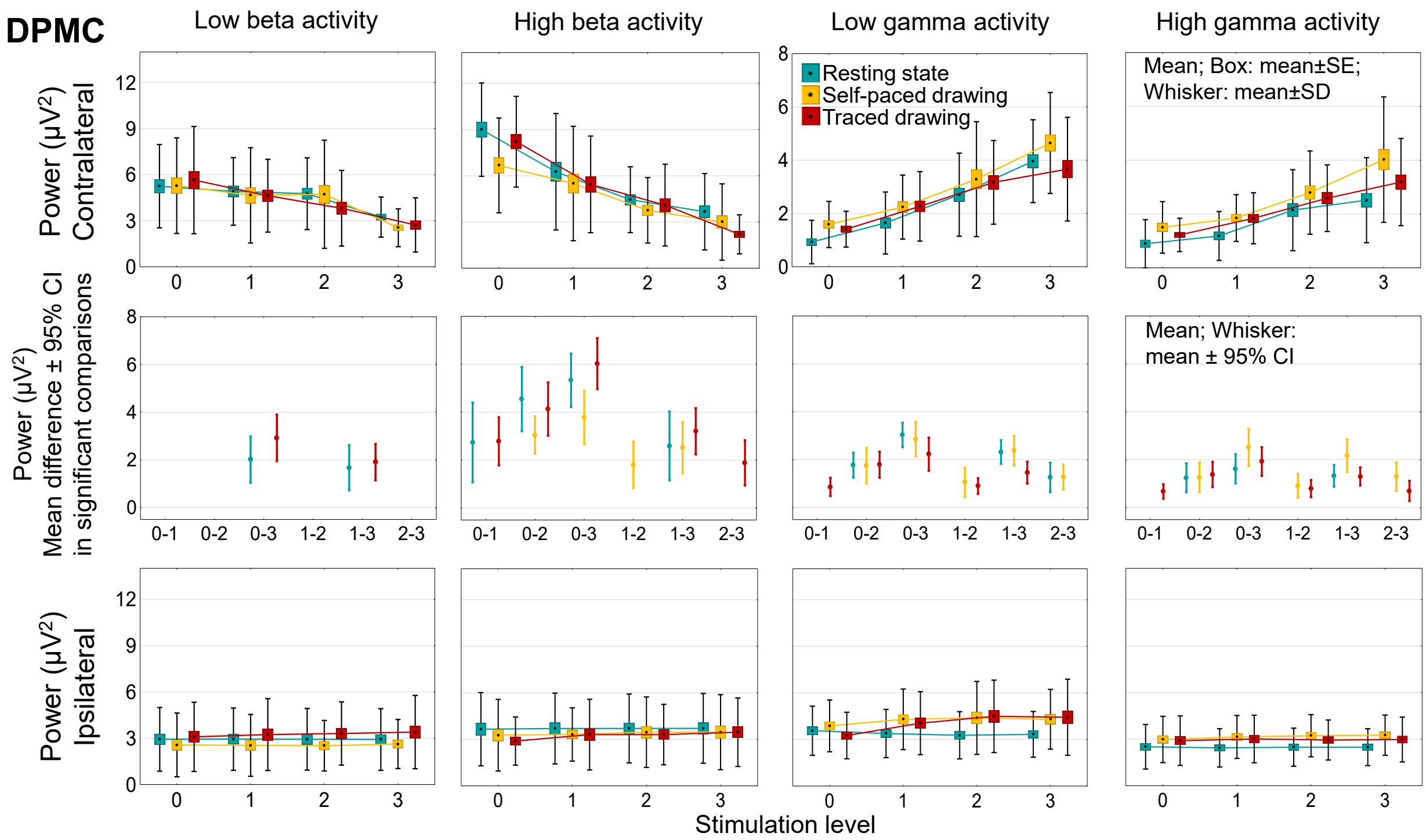

Supplementary Fig. 4. Absolute beta- and gamma-band power across stimulation intensities in the DPMC

A subset of the spiral drawing data has been published previously[1]. DPMC, dorsal premotor cortex

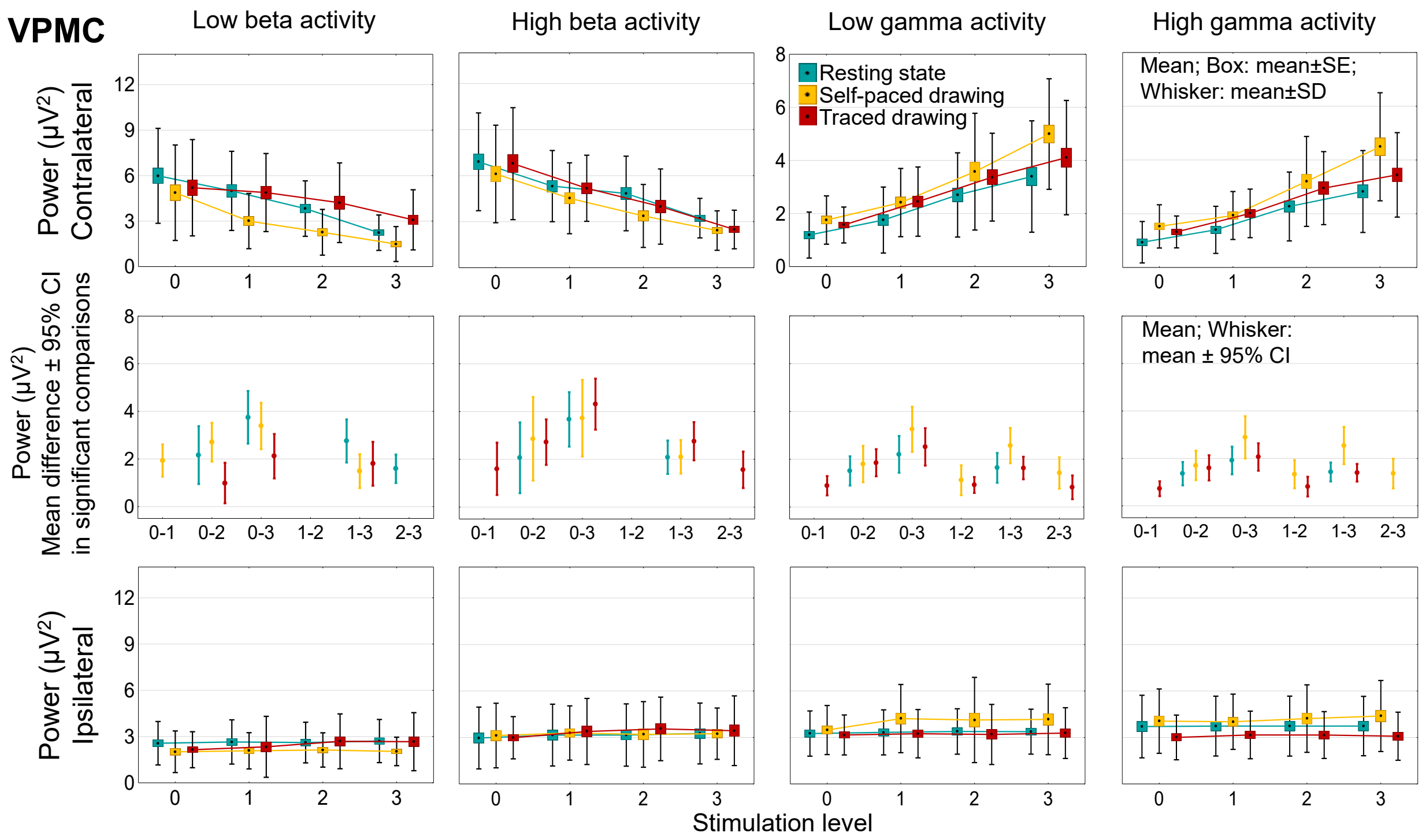

Supplementary Fig. 5. Absolute beta and gamma power changes in the VPMC

A subset of the spiral drawing data has been published previously[1]. VPMC, ventral premotor cortex

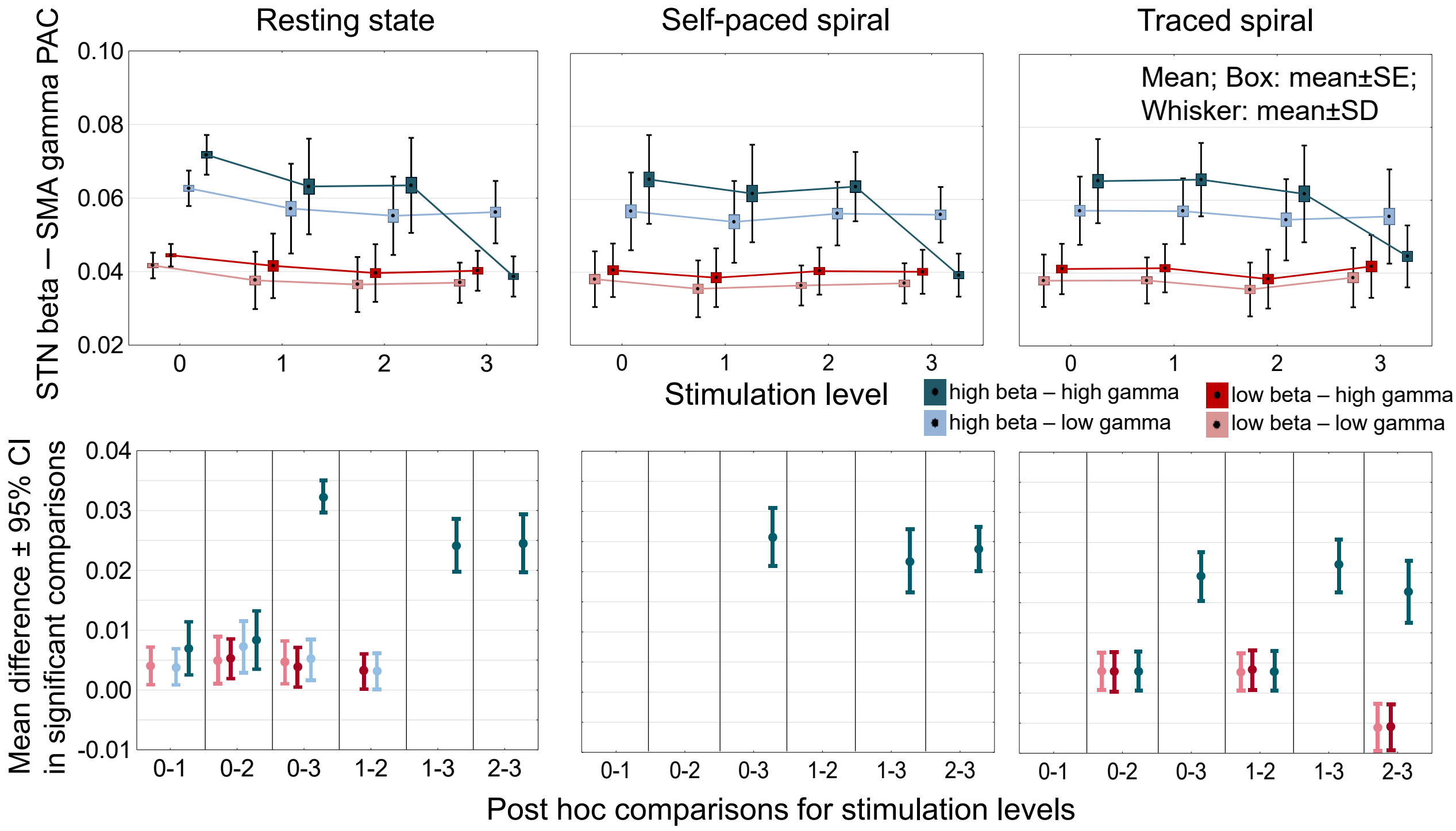

Supplementary Fig. 6. Interregional phase–amplitude coupling changes between STN beta and SMA gamma frequency band pairs

Interregional phase–amplitude coupling between the high beta activity of the STN and high gamma activity of the supplementary motor area decreased at stimulation level 3 in all tasks. In the meantime, the coupling between the STN and SMA in the high beta–low gamma, low beta–high gamma, and low beta–low gamma frequency band pairs remained unaffected by stimulation. Significant post hoc comparisons of the PAC values at the stimulation levels are presented in the bottom row. STN, subthalamic nucleus; SMA, supplementary motor cortex

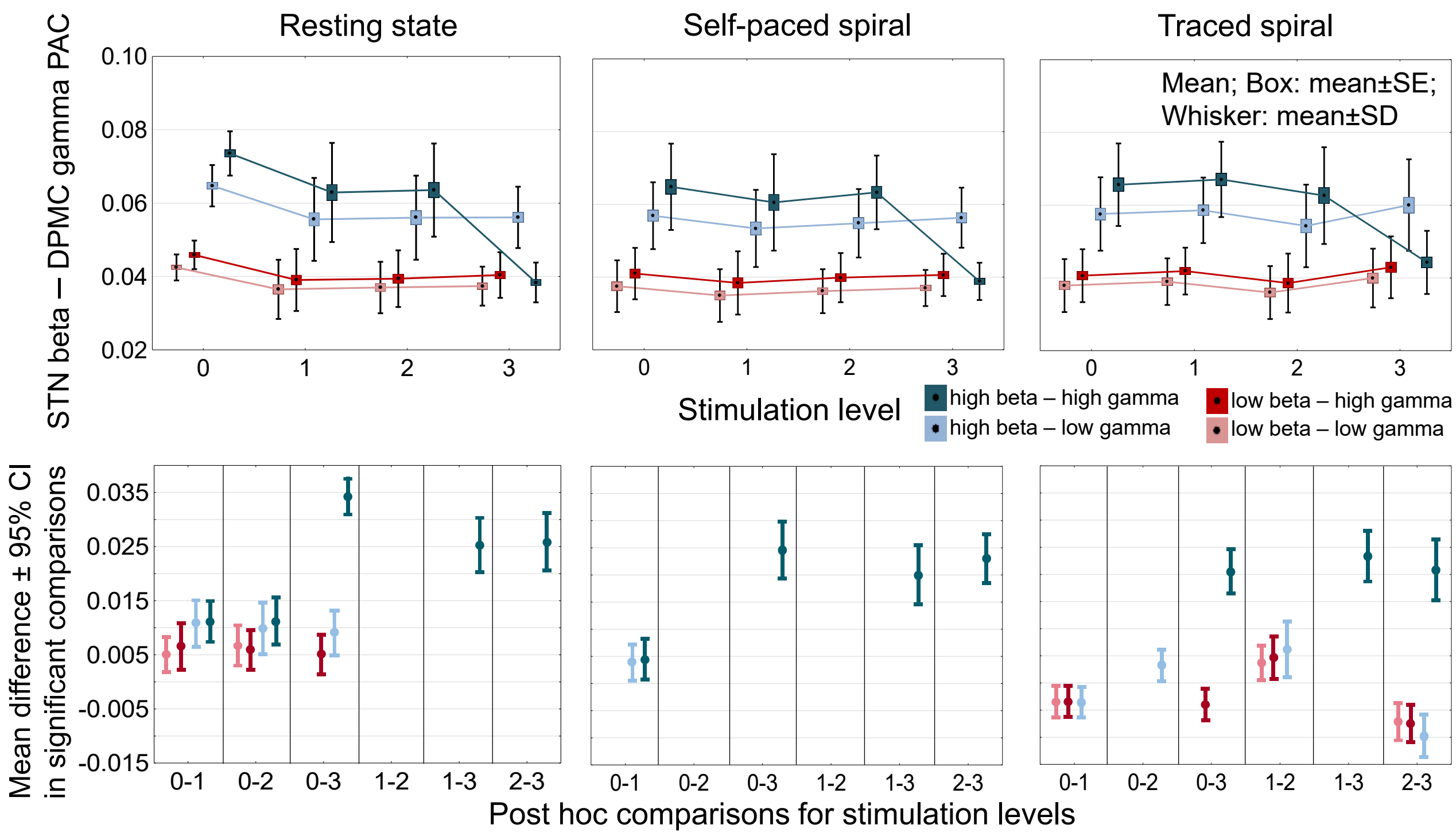

Supplementary Fig. 7. Subthalamic stimulation decreases STN high beta–DPMC high gamma interregional phase amplitude coupling

Ramping subthalamic stimulation decreased the interregional phase–amplitude coupling between the high beta activity of the STN and high gamma activity of the DPMC at stimulation level 3. STN, subthalamic nucleus; DPMC, dorsal premotor cortex

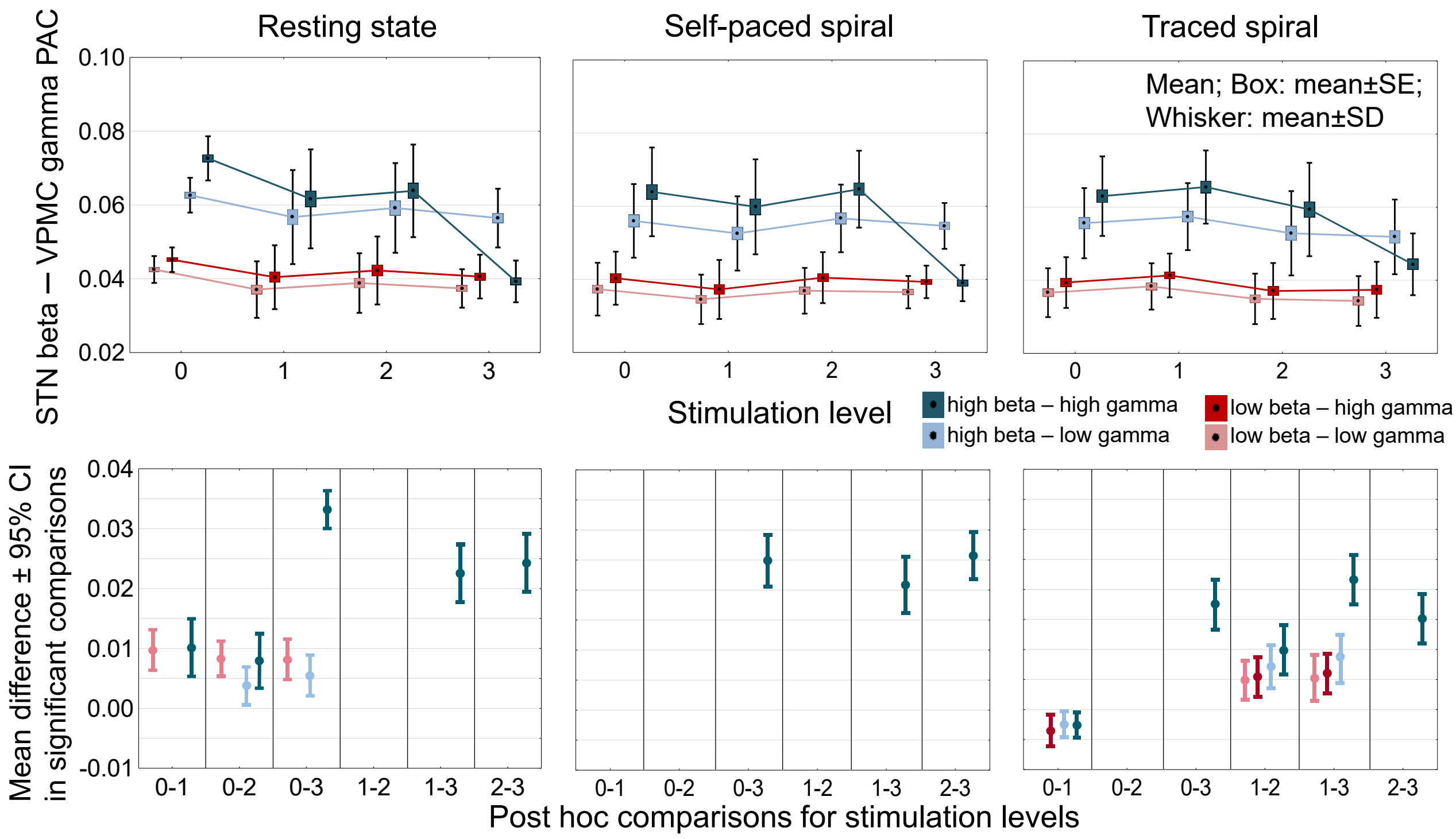

Supplementary Fig. 8. Subthalamic stimulation decreases STN high beta–VPMC high gamma interregional phase amplitude coupling

Stimulation-induced interregional phase–amplitude coupling changes between the beta activity of the STN and gamma activity of the VPMC are similar to the changes observed in the primary motor cortex, supplementary motor area, and dorsal premotor cortex (Fig. 3, Supplementary Fig. 6-7). STN, subthalamic nucleus; VPMC, ventral premotor cortex

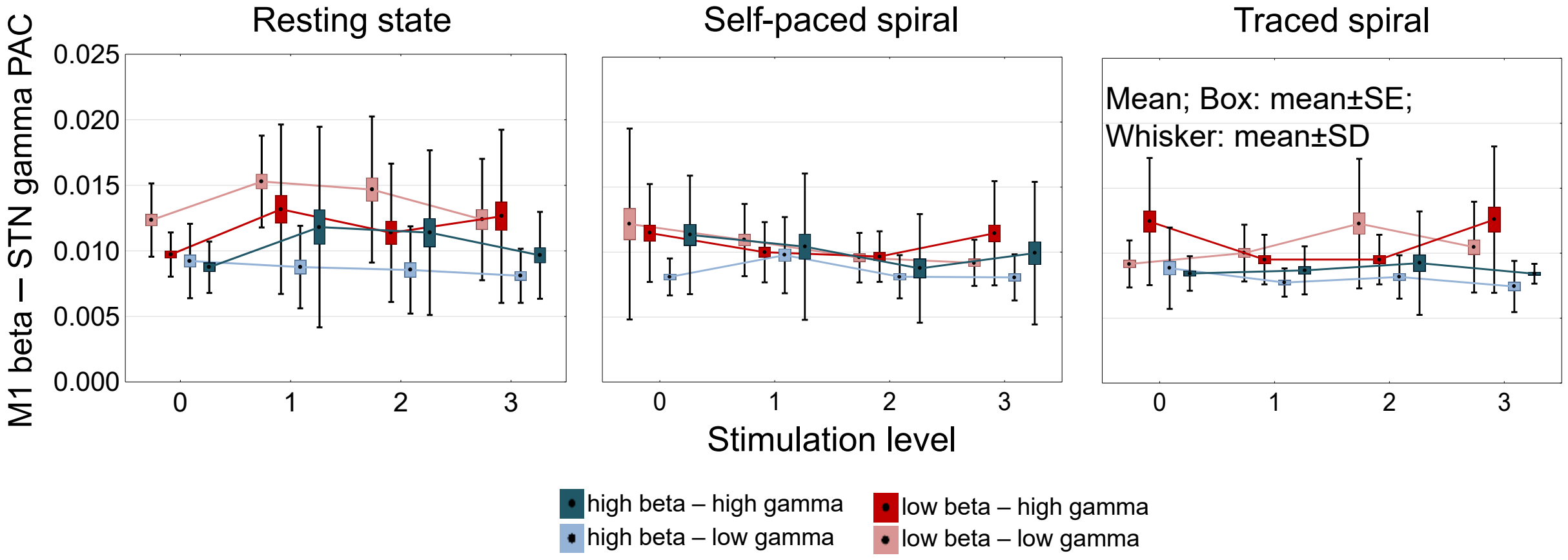

Supplementary Fig. 9. Interregional phase–amplitude coupling changes between M1 beta and STN gamma frequency band pairs

Interregional phase–amplitude coupling (PAC) between beta activity in the primary motor cortex and gamma activity in the STN did not change significantly with ramping stimulation level across all tasks and band pairs. M1, primary motor cortex; STN, subthalamic nucleus

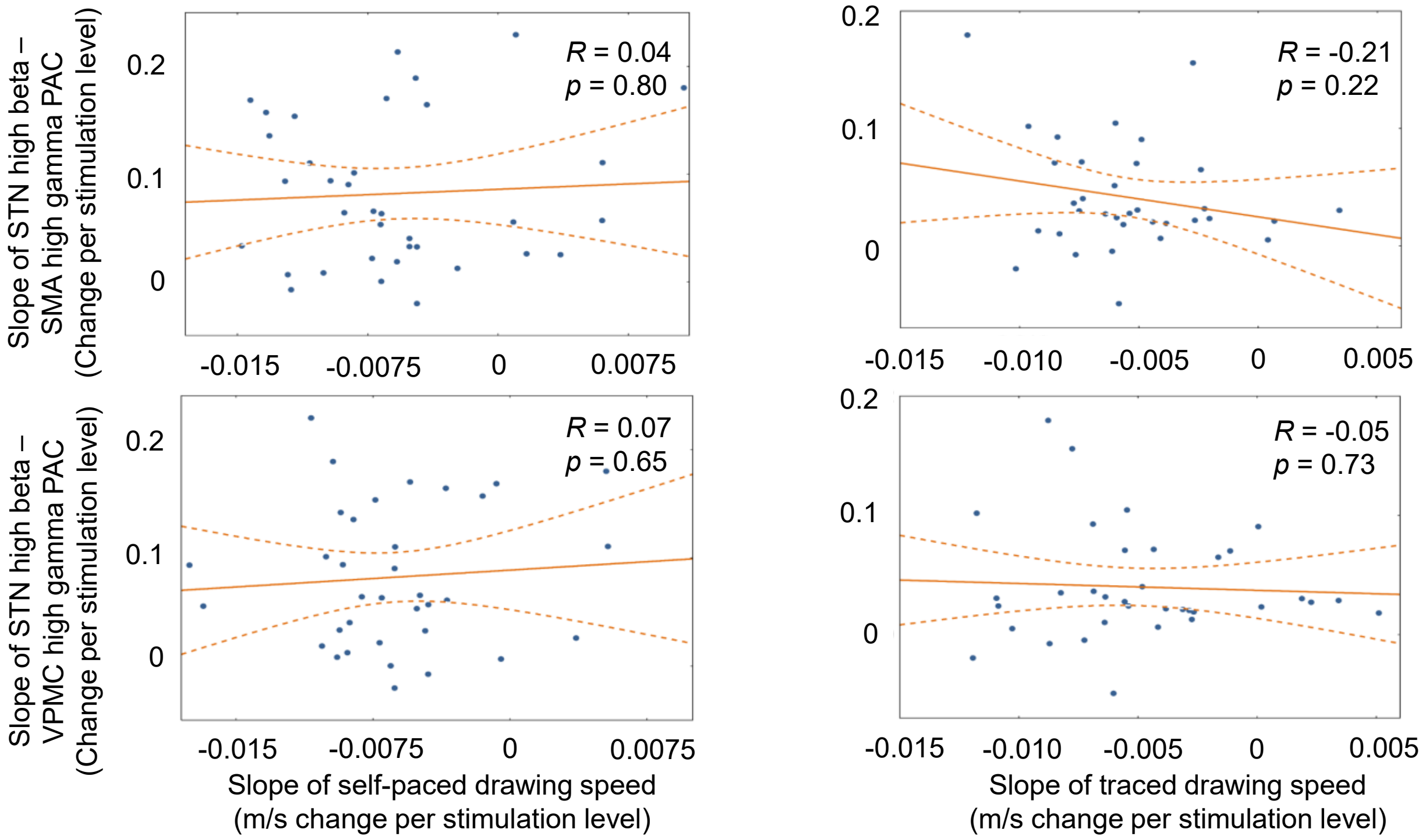

Supplementary Fig. 10. Phase–amplitude coupling and self-paced drawing speed improvement

The stimulation-induced change in PAC strength between the subthalamic high beta and high gamma band in (**A**) SMA or (**B**) VPMC did not correlate with the improvement in self-paced drawing speed. Likewise, the change in PAC strength between the subthalamic high beta and the (**C**) SMA or (**D**) VPMC high gamma band did not correlate with the improvement in traced drawing speed. PAC, phase–amplitude coupling; SMA, supplementary motor area; VPMC, ventral premotor cortex

Supplementary Table 1. Prediction of PAC with a Support Vector Regression model. Shapley Values are presented for all analyzed region pairs in self-paced and traced spiral drawings

High values of R^2 indicate a strong predictive outcome.

- Permutation test p-value (10,000 permutations): the fraction of permuted-label runs that achieved an OOF $R^2 \geq$ the observed value;

- Fisher's combined probability test ($\chi^2(16) = 62.15$, $p = 2.26 \times 10^{-7}$) was applied across all eight permutation p-values to test the global null hypothesis that none of the eight SVR models captures a true relationship between predictors and PAC suppression. The test statistic follows a χ^2 distribution with 2k degrees of freedom (k = 8 models). Three models individually survived permutation testing after applying no correction (Y-SELF-STN-M1: OOF $R^2 = 0.36$, $p < 0.0001$; Y-TRACED-STN-M1: OOF $R^2 = 0.33$, $p = 0.0006$; Y-TRACED-STN-PMC: OOF $R^2 = 0.74$, $p < 0.0001$); the remaining five targets showed no predictive signal (OOF $R^2 \leq 0$, $p > 0.17$).

**Model**	**Train R2**	**Val R2**	**OOF R2**	**p-value (Bonferroni test)**
Y-SELF-STN-M1	0.352 ± 0.041	0.212 ± 0.246	**0,3608**	**0,0008**
Y-SELF-STN-PMC	-0.070 ± 0.122	-1.143 ± 1.415	-0,0209	1
Y-SELF-STN-SMA	-0.004 ± 0.005	-0.066 ± 0.059	-0,0018	1
Y-SELF-STN-VPMC	-0.010 ± 0.013	-0.142 ± 0.109	-0,0009	1
Y-TRACED-STN-M1	0.327 ± 0.069	0.168 ± 0.337	**0,3348**	**0,0048**
Y-TRACED-STN-PMC	0.724 ± 0.102	0.453 ± 0.624	**0,7378**	**0,0008**
Y-TRACED-STN-SMA	-0.021 ± 0.035	-0.362 ± 0.489	-0,0072	1
Y-TRACED-STN-VPMC	-0.057 ± 0.052	-0.578 ± 0.879	-0,0436	1
Fisher combined (all 8 models)			chi2(16)=62.153	2,261E-07
Fisher combined (positive OOF R2 only, n=3)			chi2(6)=51.679	2,2E-09

STN, subthalamic nucleus; M1, primary motor cortex; DPMC, dorsal premotor cortex; SMA, supplementary motor area; VPMC, ventral premotor cortex; SVR, support vector regression

Localization of active contacts

DBS electrode positions were reconstructed using Lead-DBS software [2] (version 3.1, https://www.lead-dbs.org/).

Postoperative CT or MRI images were co-registered to preoperative MRI scans using Advanced Normalization Tools (ANTs; http://stnava.github.io/ANTs/) and corrected for potential intraoperative brain shift with an automated subcortical refinement module. Normalization warp fields were manually adjusted using the WarpDrive toolbox (version 3.115), part of Lead-DBS, with particular attention to any misalignments in the anatomy of the subthalamic nucleus. For visualizations and analyses, we used the STN atlas definitions from the DBS Intrinsic Template (DISTAL) atlas, version 1.1 [3]. The integration of active electrode contacts was simulated using the SimBio/FieldTrip pipeline (https://www.mrt.uni-jena.de/simbio/ and http://fieldtriptoolbox.org/) implemented in Lead-DBS, version 3.1. Euclidean distance between the active contact points and the center of the STN motor segment was calculated for each patient.

EEG source reconstruction

Source analysis was conducted as described in our previous publication [1]. We estimated a lead-field matrix using the finite-element method (FEM) to examine how the source space generates differences in scalp potentials measured with EEG electrodes. For this, a realistic head model was created with individual geometry and specific tissue conductivity values (grey matter, white matter, cerebrospinal fluid, bone, and skin) based on preoperative T1-weighted MRI of the patients. The source-space signals were estimated using a linearly constrained minimum variance (LCMV) beamforming technique, a spatial filtering method that defines the relative contribution of each dipole to the differences in electrical potentials by enhancing signals coming from a particular location while attenuating contributions from other areas. A detailed description of the inverse solution based on LCMV spatial filtering is provided elsewhere [4]. We identified the key motor areas involved in spiral drawing using the Human Motor Area Template atlas for the primary motor cortex, dorsal premotor (DPMC) and ventral premotor (VPMC) cortices and supplementary motor area [5], and the Atlas of the basal ganglia (ATAG) [6] for the subthalamic nucleus. (Fig. 2C).

[1] Berki Á J, Ding H, Palotai M, Halász L, Erőss L, Fekete G, et al. Subthalamic stimulation evokes hyperdirect high beta interruption and cortical high gamma entrainment in Parkinson's disease. NPJ Parkinsons Dis 2025;11(1):95.

[2] Neudorfer C, Butenko K, Oxenford S, Rajamani N, Achtzehn J, Goede L, et al. Lead-DBS v3.0: Mapping deep brain stimulation effects to local anatomy and global networks. Neuroimage 2023;268:119862.
[3] Ewert S, Plettig P, Li N, Chakravarty MM, Collins DL, Herrington TM, et al. Toward defining deep brain stimulation targets in MNI space: A subcortical atlas based on multimodal MRI, histology and structural connectivity. Neuroimage 2018;170:271–82.
[4] Van Veen BD, van Drongelen W, Yuchtman M, Suzuki A. Localization of brain electrical activity via linearly constrained minimum variance spatial filtering. IEEE Trans Biomed Eng 1997;44(9):867–80.
[5] Mayka MA, Corcos DM, Leurgans SE, Vaillancourt DE. Three-dimensional locations and boundaries of motor and premotor cortices as defined by functional brain imaging: a meta-analysis. Neuroimage 2006;31(4):1453–74.
[6] Keuken MC, Bazin PL, Schäfer A, Neumann J, Turner R, Forstmann BU. Ultra-high 7T MRI of structural age-related changes of the subthalamic nucleus. J Neurosci 2013;33(11):4896–900.
